# Outcomes and costs of publicly funded patient navigation interventions to enhance HIV care continuum outcomes in the United States: A before-and-after study

**DOI:** 10.1371/journal.pmed.1003418

**Published:** 2021-05-13

**Authors:** Starley B. Shade, Valerie B. Kirby, Sally Stephens, Lissa Moran, Edwin D. Charlebois, Jessica Xavier, Adan Cajina, Wayne T. Steward, Janet J. Myers

**Affiliations:** 1 Center for AIDS Prevention Studies, Department of Medicine, University of California San Francisco, San Francisco, California, United States of America; 2 Institute for Global Health Sciences, Department of Epidemiology and Biostatistics, University of California San Francisco, San Francisco, California, United States of America; 3 Independent Consultant, Silver Spring, Maryland, United States of America; 4 Health Resources and Services Administration, HIV/AIDS Bureau, Rockville, Maryland, United States of America; Boston University School of Public Health, UNITED STATES

## Abstract

**Background:**

In the United States, patients with HIV face significant barriers to linkage to and retention in care which impede the necessary steps toward achieving the desired clinical outcome of viral suppression. Individual-level interventions, such as patient navigation, are evidence based, effective strategies for improving care engagement. In addition, use of surveillance and clinical data to identify patients who are not fully engaged in care may improve the effectiveness and cost-effectiveness of these programs.

**Methods and findings:**

We employed a pre-post design to estimate the outcomes and costs, from the program perspective, of 5 state-level demonstration programs funded under the Health Resources and Services Administration’s Special Projects of National Significance Program (HRSA/SPNS) Systems Linkages Initiative that employed existing surveillance and/or clinical data to identify individuals who had never entered HIV care, had fallen out of care, or were at risk of falling out of care and navigation strategies to engage patients in HIV care. Outcomes and costs were measured relative to standard of care during the first year of implementation of the interventions (2013 to 2014). We followed patients to estimate the number and proportion of additional patients linked, reengaged, retained, and virally suppressed by 12 months after enrollment in the interventions. We employed inverse probability weighting to adjust for differences in patient characteristics across programs, missing data, and loss to follow-up. We estimated the additional costs expended during the first year of each intervention and the cost per outcome of each intervention as the additional cost per HIV additional care continuum target achieved (cost per patient linked, reengaged, retained, and virally suppressed) 12 months after enrollment in each intervention. In this study, 3,443 patients were enrolled in Louisiana (LA), Massachusetts (MA), North Carolina (NC), Virginia (VA), and Wisconsin (WI) (147, 151, 2,491, 321, and 333, respectively). Patients were a mean of 40 years old, 75% male, and African American (69%) or Caucasian (22%). At baseline, 24% were newly diagnosed, 2% had never been in HIV care, 45% had fallen out of care, and 29% were at risk of falling out of care. All 5 interventions were associated with increases in the number and proportion of patients with viral suppression [percent increase: LA = 90.9%, 95% confidence interval (CI) = 88.4 to 93.4; MA = 78.1%, 95% CI = 72.4 to 83.8; NC = 47.5%, 95% CI = 45.2 to 49.8; VA = 54.6, 95% CI = 49.4 to 59.9; WI = 58.4, 95% CI = 53.4 to 63.4]. Overall, interventions cost an additional $4,415 (range = $3,746 to $5,619), $2,009 (range = $1,516 to $2,274), $920 (range = $627 to $941), $2,212 (range = $1,789 to $2,683), and $3,700 ($2,734 to $4,101), respectively per additional patient virally suppressed. The results of this study are limited in that we did not have contemporaneous controls for each intervention; thus, we are only able to assess patients against themselves at baseline and not against standard of care during the same time period.

**Conclusions:**

Patient navigation programs were associated with improvements in engagement of patients in HIV care and viral suppression. Cost per outcome was minimized in states that utilized surveillance data to identify individuals who were out of care and/or those that were able to identify a larger number of patients in need of improvement at baseline. These results have the potential to inform the targeting and design of future navigation-type interventions.

## Abstract 2

Starley Shade and co-workers study outcomes and costs associated with interventions seeking to improve engagement with HIV care in the United States.

## Author summary

**Why was this study done?:**

➢ The Health Resources and Services Administration’s Special Projects of National Significance Program (HRSA/SPNS) funded an initiative that implemented 5 state-level interventions which used existing surveillance and/or clinical data to identify individuals who had never entered HIV care, had fallen out of care, or were at risk of falling out of care and navigation strategies to engage patients in HIV care.
➢ This study estimates the outcomes and incremental costs of these 5 state-level interventions relative to the standard of care provided prior to implementation of the interventions.

**What did researchers do and find?:**

➢ We estimated the additional cost of interventions during the first year of implementation (2013 to 2014). Among HIV–infected patients who enrolled in each intervention during the first year of implementation, we estimate how much the number and proportion of patients with viral suppression increased from before to up to 12 months after enrollment in the intervention. Then, we estimated the additional cost per additional patient with viral suppression for each intervention.
➢ We found that these interventions had similar or lower costs than similar previous interventions ($223 to $3,631 per patient), were associated with larger increases in the proportion of patients with viral suppression than similar previous interventions (47.5% to 90.9% increase in proportion of patients with viral suppression), and had similar or lower costs per additional patient with viral suppression compared to similar previous interventions ($920 to $4,415 per additional patient with viral suppression).

**What do these findings mean?:**

➢ These results show that using existing data to identify HIV–infected patients who are out of care and patient navigation to link, reengage, or retain patients in care is associated with increased viral suppression and had similar costs per outcome compared to patient navigation alone. These results also showed that use of surveillance data to identify HIV–infected patients who are out of care does not increase costs and may improve the cost per outcome of these interventions.
➢ This study did not include a contemporaneous comparison group. Therefore, we do not know the degree to which patients would have engaged or reengaged in care and achieved viral suppression due to other changes in care that occurred during the first year of implementation of the intervention.

## Introduction

The National HIV/AIDS Strategy for the United States (updated to 2020; [NHAS]) targets components for individual- and systems-level care interventions along an HIV care continuum [1]. This progression is divided into continuum categories of “identification” (testing and diagnosis), “linkage” (entering or reengaging in HIV care), “retention” (ongoing engagement in care and treatment over time), “receipt of antiretroviral therapy,” and “viral suppression” (achieving and maintaining a suppressed viral load) [2]. In the surveillance and study of HIV care and treatment outcomes, the care continuum model provides a way to understand where interventions are needed and how they should be applied across these stages of care for persons living with HIV (PLWH).

In the US, national HIV care continuum data have shown a steep drop-off after diagnosis, at the linkage and retention (cumulatively referred to as “engagement”) stages, indicating that many of those identified as being HIV–positive are not effectively engaged in care [3]. Estimates indicate that most people with HIV have been given a diagnosis (86%) but that only half of all PLWH in the US are currently retained in care (defined by federal agencies as two or more care visits, spaced at least 3 months apart, within the past 12 months) [4,5]. In 2016, the Center for Disease Control and Prevention (CDC) found that of the total number of estimated PLWH in the US (1.2 million), 59.8% were estimated to have achieved viral suppression, the desired outcome of engagement [6].

Current trends in HIV intervention design target specific stages of the HIV care continuum, and there is an increasing emphasis on the implementation of proven-effective and efficient intervention strategies [7,8]. In general, targeted HIV care and treatment interventions have been found to be effective strategies to improve linkage and retention outcomes [9–11], with some data suggesting that patient navigation-type intervention components may deliver concrete increases in patient engagement [12–14]. Despite this, much about sustainable programming and cost of interventions to enhance linkage and retention remains unknown [15].

Results from studies that include data on the cost of HIV linkage and retention initiatives in the US suggest that program implementation costs are relatively low, that cost-effectiveness and cost-savings thresholds are achievable, and that interventions targeting specific stages of the HIV care continuum are efficient investments [16–19]. Additionally, although very few navigation-type interventions have been subjected to robust economic analyses, the available research has shown navigation-type interventions, such as case management, peer navigation, and patient advocacy, to be both effective and cost-effective or cost-saving [18,20,21]. Two studies that use cost-effectiveness modeling further emphasized the strategic importance and potential impact of interventions that target retention in care. One study projected that improved retention outcomes could transform the economic trajectory of HIV and AIDS-related healthcare costs over the next 20 years, over and above what could be achieved at other stages of the care continuum [22,23]. The potential programmatic and economic value of navigation intervention components is clear, but more research is needed.

Similar to intervention strategies, the benefits of using HIV surveillance data to identify patients who are not achieving care continuum targets are generally acknowledged [1], but the outcomes and costs of using these data in the context of public health interventions are not well understood. The NHAS includes city, state, and national HIV surveillance data use in its recommended actions to intensify HIV prevention efforts, as well as in several of its policy and action steps, underscoring the broad importance of HIV surveillance data use in achieving national goals [1]. The next step in the exploration of this existing resource is to include HIV surveillance data use in economic analyses of care continuum interventions.

The outcomes and costs of HIV interventions are of particular importance for 2 reasons: (1) many HIV programs are publicly funded and must maximize desired outcomes with the input of limited resources; and (2) the advancing goal of many grant-based interventions is to move toward program integration, such that initially time-bound interventions are implemented as on-going components of core HIV care. A recent review of HIV treatment interventions has shown that measurable gains can be modest in the short term and difficult to sustain, which underscores the importance of identifying effective intervention strategies that are efficient enough to be integrated into patient care and maintained over long periods of time [24].

This paper presents results from an economic analysis of 5 patient navigation components alone (3 states) and in combination with the use of surveillance data (2 states) from 5 state-wide systems-level demonstration projects. Our analysis focuses on absolute measurable gains at the programmatic level across state health departments targeting improvements in linkage to HIV care and patient HIV viral suppression. We present the additional costs associated with implementation of these interventions and cost per outcome with respect to care continuum stages. Our analysis asked the questions: “In states attempting to develop and implement systems-level interventions, what are the outcomes and costs associated with patient navigator intervention components?”

## Methods

### Target population and setting

From 2011 to 2015, the United States Health Resources and Services Administration (HRSA), HIV/AIDS Bureau (HAB)’s Special Projects of National Significance (SPNS) conducted the Systems Linkages and Access to Care for Populations at High Risk for HIV Infection Initiative (“Systems Linkages Initiative,” or “SLI”), funding 6 state Departments of Health (DoH), over a 4-year period, with the goal of implementing interventions targeting linkage to and retention in HIV care. Researchers at the University of California, San Francisco (UCSF) were additionally funded as the Evaluation and Technical Assistance Center (ETAC) to conduct a cross-state evaluation and provide technical assistance across demonstration states.

The broad goal of SLI was to create effective and sustainable linkages to care across systems within demonstration states using a tailored set of strategies that harnessed and coordinated components of the existing HIV healthcare infrastructure. Strategies included disease surveillance, patient engagement, and care and support services including navigation. The interventions are described in detail elsewhere [25]. Specifically, the initiative aimed to target individuals who had been identified as HIV–positive but were either not engaged in care, suboptimally engaged in care, or were no longer engaged in (had fallen out of) care.

A condition of funding was that the first 2 years be spent designing and piloting interventions in Learning Collaboratives based on the Collaborative Model, a program strategy designed by the Institute for Healthcare Improvement that pursues twin goals of service delivery quality improvement and health organization cost reduction [26]. Each state Department of Health (DoH) engaged in collaborations with external agencies that specifically served or interacted with communities vulnerable to HIV, such as HIV testing facilities and clinics, correctional facilities, or community-based organizations. These organizations participated in the Learning Collaborative model and developed systems-level interventions that were implemented in the last 2 years of the project. We did not have a published protocol for this analysis because the interventions emerged as part of the Learning Collaborative. However, plans for data collection and analysis of costs and cost per HIV care continuum outcome were presented to States in advance of data collection (S1 Text). This plan did not specify methods for sensitivity analysis.

### Interventions

Across states, 16 interventions were selected for implementation from the Learning Collaboratives. While themes (such as ensuring cultural tailoring) existed across the intervention approaches, significant diversity persisted, which precluded an overall analysis of all interventions. Five interventions in Louisiana (LA), Massachusetts (MA), North Carolina (NC), Virginia (VA), and Wisconsin (WI) were selected for assessment of outcomes and costs according to the Consolidated Health Economic Evaluation Reporting Standards (CHEERS) statement (S1 CHEERS Checklist) because they used existing data (including surveillance data) to identify patients who were not fully engaged in care AND incorporated aspects of patient navigation to engage patients in HIV care [27]. Table 1 provides brief description of each of the 5 interventions included here. Detailed information on 4 of 5 of these interventions has been previously published [28–31]. A protocol for the fifth is included in the Supporting information (S2 Text).

**Table 1:** Descriptions of 5 navigator-like interventions selected for cost analysis.

State	Implementation Dates	Identification criteria	Intervention description	Exit criteria
LA	1 October 2013–31 October 2014	Participating prisons kept lists of PLWH in custody, generated using pharmacy and laboratory records. Incarcerated PLWH on those lists with a scheduled release date within 180 days were considered eligible.	Interventionists helped clients select an ASO and conducted a video conference with the ASO case manager and the client to plan for connection to and retention in medical care following release.	Upon release, clients either met with an ASO case manager within 4 weeks or were prompted for up to 12 weeks to do so.
MA	27 March 2013–26 March 2014	Clients were identified through provider recommendation, acuity scales, and out-of-care lists generated using statewide HIV surveillance data. Eligible PLWH were: newly diagnosed; recent immigrants; recently incarcerated; out of care; or, at high risk of falling out of care.	Teams of nurses and HIV–positive peers provided 6–12 months of intensive linkage and retention support, including medical case management, home visits, medical visit accompaniment, assessment of barriers to care, and the development of individualized care plans.	Teams regularly completed an acuity scale using clinic-level and state surveillance data and reviewed patient service and treatment plans. Services were discontinued at 6, 9, or 12 months depending on acuity and service plan progress.
NC	1 March 2013–28 February 2014	Program 1 used state-level surveillance reports to identify PLWH who had not had an appointment in ≥6–9 months. Program 2 targeted individuals who could not be reached by Program 1 or did not reengage in care; or, were newly diagnosed, pregnant and out of care, or recently released from prison, as identified by disease intervention specialists or corrections personnel.	Program 1 regularly reviewed state-level surveillance data to identify PLWH who were out of care, and then attempted for 30 days to contact and reengage these individuals. Individuals who could not be reached were referred to Program 2. Program 2 counselors used strengths-based counseling and an assessment of barriers to care to support linkage and retention.	Program 1 enrollment discontinued after a client attended an HIV medical appointment or after 30 days. Program 2 enrollment discontinued after a client attended an HIV medical appointment or at 90 days. Appointment attendance was recorded by interventionists or monitored through using medical and surveillance records.
VA	1 September 2013–31 August 2014	PLWH who were newly diagnosed, out of care, or at risk of falling out of care were eligible. Referrals were made by ASOs, medical providers, local health departments, disease intervention specialists, and HIV case managers.	Navigators used client-centered counseling and motivational interviewing to identify barriers to care. Navigators also facilitated connection to medical providers, social services, and community resources, and provided HIV health education.	Navigators and clients regularly completed a form assessing medical appointment attendance, barriers to care, and medication pickup. Services continued for 3–12 months depending upon the results of this assessment.
WI	1 June 2013–31 May 2014	PLWH who were newly diagnosed, out of care, recently incarcerated, or at risk of falling out of care were referred by testing and partner services, HIV clinics and ASOs, corrections personnel.	Interventionists provided intensive linkage, retention, and care coordination support for up to 9 months, including frequent contact, assessment of barriers to care, and referral to support services.	Services were provided until the individual attended 3 HIV medical care appointments, or for up to 9 months. As needed, individuals were transitioned to case management.

ASO, AIDS service organization; LA, Louisiana; MA, Massachusetts; NC, North Carolina; PLWH, persons living with HIV; VA, Virginia; WI, Wisconsin.

### Patients

We measured the outcomes of each patient navigation intervention among patients enrolled during the first 12 months of implementation (interventions started between March 2013 and October 2013). Patients were followed from enrollment until they exited the intervention or until 12 months after enrollment. State DoHs submitted individual-level deidentified data to the ETAC on a quarterly basis. These data included information about patient characteristics at enrollment: age; gender (male, female, transgender); race/ethnicity (African American, white, Latinx/Hispanic, other/mixed race/ethnicity); insurance status (private, Medicare, Medicaid, other public insurance, no insurance, or other/unknown insurance status); HIV risk category (heterosexual sex, injecting drug use, male who has sex with men, other or unknown risk); CD4 cell count at diagnosis; and engagement in HIV care prior to enrollment (those who had never received HIV care–not linked; those who were not currently engaged in HIV care–not engaged; those who were currently engaged in HIV care, but at risk of falling out of care due to identified risk factors of patient acuity, homelessness, and drug use–in care/at risk). These data also included information on: medical visit or lab records showing receipt of primary HIV care; and viral load test results.

### Outcomes

To examine the outcomes of these patient navigation interventions in linking, retaining, and reengaging patients, we reviewed the following measures of engagement in HIV care both at the time of patient enrollment (assessed up to 24 months prior to enrollment) and achievement of each outcome (up to 12 months after enrollment): “linkage”–ever having received HIV care; “reengagement”–any receipt of HIV care after enrollment among patients who were out of care at enrollment; “retention”–one primary HIV care visit in each of the past two 6 months’ time intervals separated by 60 days; and “viral suppression”–last viral load test result in time period less than 200 copies/mL. For our primary or, base case analyses, individuals who were released from jail or prison were categorized as being out of care at enrollment based on the assumption that release from jail or prison is equivalent as having an interruption in care. Individuals who had not previously received HIV care (not linked) or were out of care at enrollment were assumed to be not engaged in care and not virally suppressed.

### Costs

We estimated the incremental costs, from the program perspective, of the first year of implementation for these 5 navigation-type interventions relative to standard of care. Costs included actual costs of program implementation including both HRSA SPNS program expenditures and in-kind contributions from other sources. Interventions were assumed to sit on top of existing infrastructure and services. We did not have the resources to estimate the costs of existing infrastructure and services. Therefore, the costs of implementation of the interventions were assumed to equal the incremental costs of the interventions above standard of care. Costs were estimated at 2013 USD. We did not discount costs as they were collected over a single year. However, we did amortize costs of equipment over its useable life.

Data were retrospectively captured at the end of the first year of implementation. Prior to data collection, we conducted telephone interviews with each of the 5 demonstration states to become familiar with each intervention. We then worked with states to develop a standardized Excel-based cost data collection tool (S1 Data) to collect activity-based costs across all 5 interventions. The final data collection tool captured information on: source (HRSA SPNS funds, other in-kind resources); resource category (personnel, recurring costs, capital investments, and infrastructure); time period (preimplementation or start-up, implementation); and personnel activity (referral and enrollment, contact attempts, direct service–client contact, direct service–no client contact, non-client activity, and on-site supervision and management). For personnel activities focused directly on individual patients (contact attempts, direct service–client contact, direct service–no client contact), we asked states to allocate personnel effort across select HIV care continuum targets (linkage, retention, reengagement, and viral suppression) based on the HIV care goal of each activity. Costs associated with use of state surveillance data to identify patients eligible for each intervention were included, but the costs of development and operations of state-level surveillance programs were not included, as state HIV surveillance programs were assumed to be preexisting, separate functional entities. Costs pertaining to management and evaluation of each intervention as a demonstration project (such as time related to preparing reports to HRSA SPNS) were excluded.

The strategies used by the 5 demonstration states to gather and submit the requested cost data depended on the structure and recordkeeping of each demonstration state team. For example, some of the demonstrations were led by teams comprised of state employees who had retained their own records of costs and personnel time, while others were led by teams at universities and other external entities who were contracted by the state and therefore had to request information on expenditures from state employees in charge of managing their grant. To gather the necessary information, all states had to involve finance personnel familiar with how resources were used, and program personnel familiar with how the intervention functioned.

### Analysis

Using the patient data provided by the states, we present patient characteristics by state. We estimate the number and percent of additional patients linked, reengaged, retained, and virally suppressed by 12 months after enrollment by comparing the number and proportion of patients linked, reengaged, retained, and virally suppressed overall and by state at enrollment and at the time of achievement of each outcome or end of follow-up (12 months). We computed the 95% confidence interval (CI) for number and proportion of additional patients achieving each outcome assuming a binomial distribution [32]. We employed inverse probability weighting to adjust for differences in the distribution of patient characteristics across interventions, missing data at enrollment, and loss to follow-up before 12 months without achievement of outcomes (S1 Table) [33]. Briefly, we used a series of logistic regression models to estimate the probability of being from each state, of having a missing viral load at baseline and of being lost to follow-up before 12 months and without achievement of suppressed viral load for each individual. Each model included patient characteristics as defined in S2 Table (age, gender, race/ethnicity, insurance status, HIV risk category, and CD4+ T cell count at diagnosis). The predicted probabilities from these models were used to generate 3 separate weights for each individual which were multiplied together to get a final weight. We have successfully employed this approach in evaluation of other HRSA SPNS demonstration programs [34]. From the states’ cost data, we estimated the additional programmatic costs associated with patient identification and navigation during the first year of implementation of each intervention, overall and by category (source, resource category, time period, and activity). We estimated the cost per outcome of each intervention as the additional cost per additional HIV care continuum target achieved (cost per patient linked, reengaged, retained, and virally suppressed). We conducted sensitivity analyses to identify which cost categories had the most impact on cost per outcome. We also examined the impact of the assumption that individuals who were released from prison were out of care at enrollment by comparing our observed results to those from previous literature (reengagement post-release at between 34.0% at 30 days and 85.7% at 1 year and viral suppression among those who reengage at between 71.1% at 30 days and 40.2% at 1 year) [35].

### Ethical approval

Evaluation of the Systems Linkages project was approved by the UCSF Internal Review Board. However, the costing component was deemed to be nonhuman subjects**’** research because the data related strictly to an organization and not to human participants.

## Results

### Participant enrollment

Overall, 3,443 patients were enrolled in 5 states (range = 147 to 2,491; S2 Table). Viral load data were missing at baseline for 382 patients (11.1%) and 599 patients (17.4%) did not achieve viral suppression and were lost to follow-up prior to 12 months of follow-up. As a result, we were able to evaluate change in the number and proportion of viral suppression among 2,452 participants (71.2%).

### Participant characteristics

Overall, participants were a mean of 40 years old, 75% male, and majority African American (69%) or Caucasian (22%). At enrollment, 24% were newly diagnosed, 2% had never been in HIV care, 45% had fallen out of care, and 29% were at risk of falling out of care. We are not able to ascertain insurance status for most participants (78%). However, among those for whom insurance status was reported, most (77%) had publicly funded insurance. Patient characteristics varied across states (S2 Table). We employed inverse probability weighting in subsequent analysis to adjust for differences in participant characteristics, missing data at enrollment, and due to loss to follow-up before 12 months without achievement of outcomes.

### Outcomes

**Fig 1:**
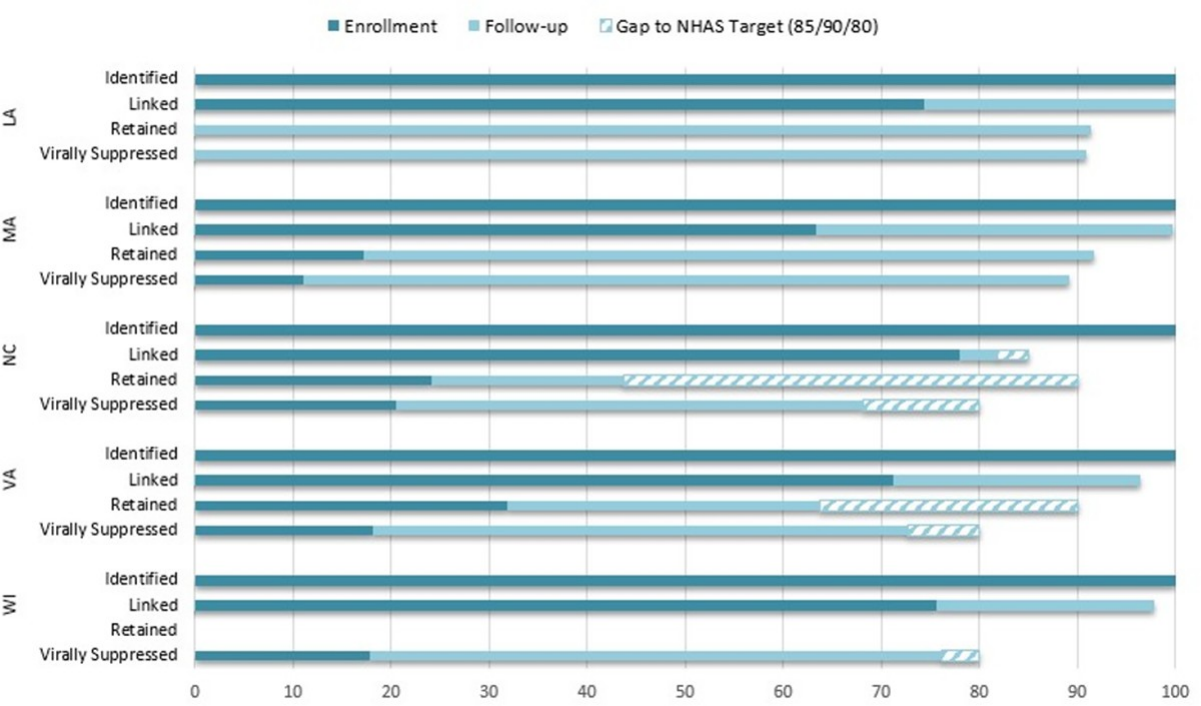
Patient navigation interventions: Estimated HIV care continuum outcomes at follow-up. LA, Louisiana; MA, Massachusetts; NC, North Carolina; NHAS, National HIV/AIDS Strategy; VA, Virginia; WI, Wisconsin.

Although most patients had been linked to care prior to enrollment (range = 63.3% to 78.0%), the proportion of participants engaged in care (range = 0.0% to 46.8%), retained in care (range = 0.0% to 31.8%) and with viral suppression (range = 0.0% to 20.5%) prior to enrollment was quite low across all states. Implementation of the interventions was associated with higher numbers of patients linked (*n =* 570.4), reengaged (*n* = 1,523), retained (*n* = 623.6), and achieving viral suppression (*n* = 1,081.9), relative to baseline, across each HIV care continuum target by 12 months after enrollment. We observed statistically significant increases in the number and proportion of enrolled patients linked (range = 37.8 to 333.2, 13.9% to 36.4%), reengaged (range = 94.7 to 1,052.5, 66.3% to 90.2%), retained (range = 64.1 to 335.5, 19.6% to 91.4%), and with viral suppression (range = 95.3 to 603.1, 47.5% to 90.8%) across all 5 interventions (Table 2). Implementation of the intervention in NC was associated with higher numbers of patients linked, reengaged, retained, and achieving viral suppression that other interventions (followed by VA and WI). Implementation of the intervention in MA was associated with higher increases in the proportion of patients linked and reengaged (followed by LA), and implementation of the intervention in LA was associated with higher increases in the proportion of patients retained and with viral suppression (followed by MA). Fig 1 highlights the extent to which these results contributed to achieving the goals of the NHAS for each demonstration state. LA and MA were able to achieve NHAS (85/90/80) targets for all HIV care continuum outcomes, while VA and WI were able to achieve the NHAS target for linkage to care.

**Table 2:** Estimated number and percent additional patients linked, reengaged, retained, and virally suppressed by 12 months (using inverse probability weighting to adjust for differences in patient populations and missingness).

State Outcome	N	Outcome at Enrollment n (%)		Outcome at Last Visit n (%)		Additional Patients n (95% CI)			Percent Additional Patients % (95% CI)		
**LA**											
**Linkage**	147	109.2	74.3%	147.0	100.0%	37.8	22.1	53.5	25.7	15.1	36.1
**Reengagement**	145	0.0	0.0%	122.7	85.8%	122.7	118.5	126.8	85.8	82.9	88.7
**Retention**	145	0.0	0.0%	132.6	91.4%	132.6	129.2	136.0	91.4	89.1	93.8
**Viral Suppression**	**131**	**0.0**	**0.0%**	**120.9**	**90.9%**	**120.9**	**117.5**	**124.2**	**90.9**	**88.4**	**93.4**
**MA**											
**Linkage**	150	95.0	63.3%	149.7	99.8%	54.7	39.7	69.6	54.7	39.7	69.6
**Reengagement**	105	0.0	0.0%	94.7	90.2%	94.7	91.6	97.7	90.2	87.3	93.1
**Retention**	86	14.8	17.2%	78.9	91.7%	64.1	57.4	70.9	74.6	66.7	82.4
**Viral Suppression**	**122**	**13.6**	**11.1%**	**113**	**89.2%**	**95.3**	**88.4**	**102.2**	**78.1**	**72.4**	**83.8**
**NC**											
**Linkage**	2,397	1,870.3	78.0%	2,203.5	81.9%	333.2	269.7	396.7	13.9	11.3	16.5
**Reengagement**	1,587	0	0.0%	1052.5	66.3%	1,052.5	1,033.7	1,071.3	66.3	65.1	67.5
**Retention**	1,710	413.0	24.2%	748.5	43.8%	335.5	302.4	386.6	19.6	17.7	21.6
**Viral Suppression**	**1,269**	**260.6**	**20.5%**	**863.7**	**68.1%**	**603.1**	**574.1**	**632.0**	**47.5**	**45.2**	**49.8**
**VA**											
**Linkage**	303	215.7	71.2%	292.2	96.4%	76.5	54.4	98.6	25.3	18.0	32.5
**Reengagement**	164	0	0.0%	123.4	75.2%	123.4	117.8	128.9	75.2	71.9	78.6
**Retention**	287	91.3	31.8%	182.7	63.7%	91.4	75.8	107.1	31.9	26.4	37.3
**Viral Suppression**	**222**	**40.3**	**18.2%**	**161.6**	**72.8%**	**121.3**	**109.7**	**133.0**	**54.6**	**49.4**	**59.9**
**WI**											
**Linkage**	308	232.8	75.6%	301.1	97.8%	68.2	45.5	91.0	22.2	14.8	29.6
**Reengagement**	164	0	0.0%	129.7	79.1%	129.7	124.5	134.9	79.1	75.9	82.3
**Retention**	NA										
**Viral Suppression**	**242**	**43.0**	**17.8%**	**184.3**	**76.2%**	**141.3**	**129.3**	**153.3**	**58.4**	**53.4**	**63.4**

CI, confidence interval; LA, Louisiana; MA, Massachusetts; NA, Not Applicable; NC, North Carolina; VA, Virginia; WI, Wisconsin.

### Costs

Table 3 shows the incremental costs associated with the first year of implementation of the patient navigation interventions. LA, NC, and WI devoted considerable resources to these interventions, while MA and VA devoted fewer resources to their programs. The source of funds for the patient navigation interventions varied across states. LA, MA, and WI utilized primarily resources from the SLI, while NC and VA contributed substantial resources from existing sources. Overall, the cost of patient navigation interventions was driven by personnel expenses. The majority of personnel resources across states were devoted to implementation of the patient navigation interventions, although LA devoted substantial resources to preimplementation (start-up) activities and LA and VA devoted substantial resources to management/oversight. Personnel costs associated with implementation of the patient navigation interventions further show that the majority of personnel activities were targeted toward individual patients (contact attempts, direct intervention, indirect intervention), rather than system-level processes (referral and enrollment) and general program management (non-client-specific activities, on-site supervision).

**Table 3:** Patient navigation interventions: Costs across funding sources, resource categories, aspects of implementation and implementation activities.

	LA		MA		NC		VA		WI	
	$	%	$	%	$	%	$	%	$	%
**Total Costs**	533,800.16	100	191,431.01	100	555,141.89	100	268,308.89	100	522,842.63	100
**Costs Across Funding Sources**										
SPNS	430,426.12	19	168,515.48	88	55,136.91	10	160,684.38	60	374,016.76	72
Existing sources	103,374.04	81	22,915.53	12	500,004.98	90	107,624.51	40	148,825.86	28
**Costs Across Resource Categories**										
Personnel	358,370.12	67	123,045.53	64	435,844.31	79	237,867.14	89	392,921.80	75
Recurring costs	10,272.69	2	360.00	0	61,837.60	11	15,230.00	6	34,949.48	7
Capital investments	64,850.49	12	2,100.00	1	29,192.18	5	9,040.00	3	7,161.31	1
Facilities	100,306.86	19	65,925.48	34	28,267.80	5	6,171.75	2	87,810.04	18
**Personnel Costs by Aspect of Implementation**										
Preimplementation	105,078.67	29	0.00	0	28,994.76	7	0.00	0	9,978.35	3
Implementation	149,570.22	42	119,858.03	97	392,034.22	90	200,840.76	69	339,370.87	86
Management/Oversight	103,721.23	39	3,187.50	3	14,815.33	3	91,830.34	31	43,137.09	11
**Implementation Costs by Activities (Personnel Costs)**										
Referral and enrollment	34,544.22	23	9,592.12	8	54,374.69	14	18,619.69	9	26,724.62	8
Contact attempts	7,186.49	5	15,725.69	13	71,374.10	18	10,364.96	5	19,230.25	6
Direct intervention	16,619.92	11	34,442.15	29	64,437.24	16	24,826.26	12	145,160.27	43
Indirect intervention	38,606.10	26	28,994.24	24	59,856.88	15	125,124.33	62	71,757.17	21
Non-client-specific	19,929.36	13	22,904.22	19	14,769.71	4	21,905.52	11	33,397.81	10
On-site supervision	32,684.14	22	8,199.61	7	127,221.60	33	0.00	0	43,100.76	13

LA, Louisiana; MA, Massachusetts; NC, North Carolina; SPNS, Special Projects of National Significance; VA, Virginia; WI, Wisconsin.

### Cost per outcome

Table 4 includes information on incremental costs and incremental cost per patient associated with each intervention, the number of additional participants who achieved each target, and incremental costs per additional HIV care continuum target achieved for each patient navigator intervention during the first year of implementation. Overall, these patient navigation interventions cost an average of $414,307 (range = $191,431 to $533,800) and $602 (range = $223 to $3,631) per patient enrolled during the first year of implementation. The overall incremental cost per additional HIV care continuum target achieved was $3,632 (range = $1,666 to $14,122) for linkage, $1,360 (range = $503 to $4,350) for reengagement, $3,322 (range = $1,655 to $4,026) for retention, and $1,915 for achievement of viral suppression. NC had the lowest cost per outcome ($920 per additional patient virally suppressed), followed by MA, VA, WI, and LA ($2,009, $2,212, $3,700, and $4,415 per additional patient virally suppressed, respectively).

**Table 4:** Cost per outcome of patient navigation interventions.

	LA		MA		NC		VA		WI		Overall	
	$	%	$	%	$	%	$	%	$	%	$	%
**Total Costs**	$533,800		$191,431		$555,142		$268,309		$522,843		**$2,071,525**	
**Cost per patient**	$3,631		$1,268		$223		$835		$1,570		**$602**	
**Intervention Outcomes (Estimated number of additional patients who achieved outcomes)**												
Linked	37.8		54.7		333.2		76.5		68.2		**570.4**	
Reengaged	122.7		94.7		1,052.5		123.4		129.7		**1,523.0**	
Retained	132.6		64.1		335.5		91.4		NA		**623.6**	
Virally Suppressed	120.9		95.3		603.1		121.3		141.3		**1,081.9**	
**Cost per Outcome (Total cost per additional patient with outcome)**												
$/Linked	$14,122		$3,500		$1,666		$3,507		$7,666		**$3,632**	
$/Reengaged	$4,350		$2,021		$503		$2,174		$4,031		**$1,360**	
$/Retained	$4,026		$2,986		$1,655		$2,936		NA		**$3,322**	
$/Viral Suppression	$4,415		$2,009		$920		$2,212		$3,700		**$1,915**	

LA, Louisiana; MA, Massachusetts; NA, Not Applicable; NC, North Carolina; VA, Virginia; WI, Wisconsin.

### Sensitivity analyses

**Fig 2:**
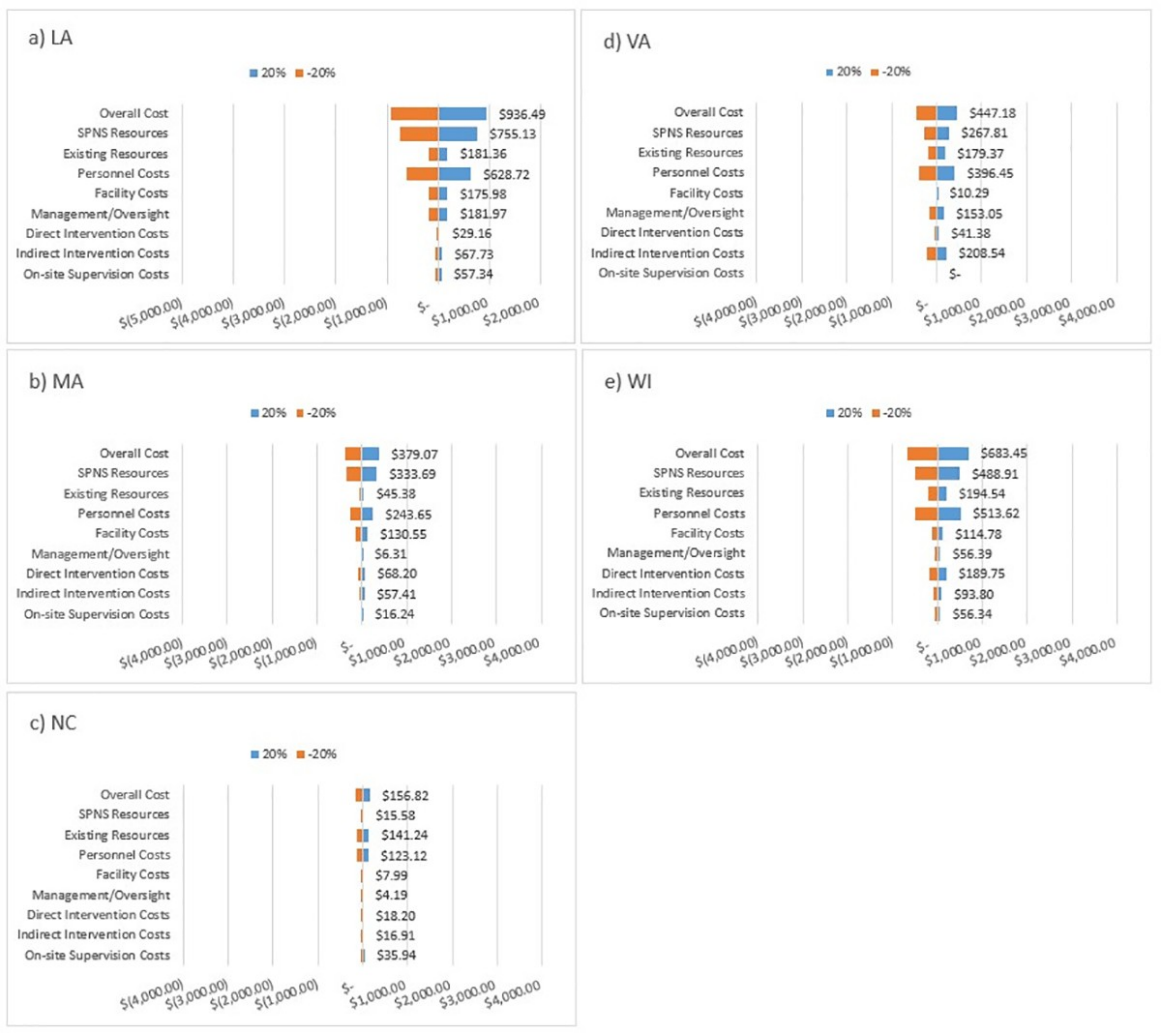
Sensitivity analyses: Effect of fluctuation in costs on the cost per outcome of patient navigation interventions. LA, Louisiana; MA, Massachusetts; NC, North Carolina; SPNS, Special Projects of National Significance; VA, Virginia; WI, Wisconsin.

We conducted sensitivity analyses to assess the effect of varying types of costs on cost per additional patient with viral suppression (Fig 2). The overall dollar amount of variation was greater for less efficient interventions. Percent variation in cost per additional patient with viral suppression was primarily driven by personnel costs and source of funding. Varying personnel costs by 20% changed cost per additional patient with viral suppression from 13% to 18% across all states. Similarly, varying the amount of HRSA SPNS resources by 20% changed costs per additional patient with viral suppression by 12% to 18% in LA, MA, and WI and changing the amount of existing resources by 20% changed cost per additional patient with viral suppression by 8% to 18% in NC and VA. Other contributors to variability in cost per outcome in specific states include: MA–facility costs; VA–management/oversight costs and indirect intervention costs (time spent on behalf of participants); and WI–direct intervention costs (time spent with participants).

## Discussion

The results of this analysis suggest that using patient navigation to engage individuals in HIV care and treatment may be associated with increases in the proportion of patients with viral suppression in states with the capacity to use existing data to identify PLWH who are not fully engaged in HIV care (those who are not linked to care and those who had fallen out of care). Each of the 5 interventions was associated with statistically significant increases in the number and percentage of patients virally suppressed after navigation services (the percent of patients virally suppressed at 12 months increased 48% to 90%). These interventions may contribute to states reaching NHAS targets for viral suppression. Although not every state achieved 80% viral suppression (per NHAS targets) as a result of their interventions, LA and MA met and exceeded this target, VA and WI came within 10% of target achievement, and NC came within 15%. The results of this study exceed those of most previous studies of the effect of patient navigation on viral suppression. In a recent review of patient navigation interventions [36], the proportion of patients with viral suppressed increased between 0% and 58%. Only one intervention showed larger increases in the proportion of patients virally suppressed than those observed in this initiative [13]. This intervention provided not only patient navigation but also intensive case management and emergency stabilization funds to support engagement in HIV care. These results add to the existing literature which demonstrates that navigation interventions can improve engagement in HIV care and subsequent viral suppression.

These results also demonstrate that use of surveillance data to identify PLWH who are not fully engaged in HIV care does not substantially increase the cost of navigation programs. Referral and enrollment comprised 8% to 23% of cost across the 5 interventions and comprised 8% and 14% of costs for the 2 interventions that utilized surveillance data to identify patients who were not fully engaged in HIV care. These findings are substantiated by previous research which found that the costs to payers of linkage and retention programs range between $1,088 and $5,447 per patient [16]. All interventions included in this initiative, including interventions in MA and NC which utilized surveillance data to identify individuals in need of servicers, fell within or below this range of previous programs.

We observed cost per outcome, measured as the incremental cost per additional patient virally suppressed, among each of the 5 patient navigation interventions. Cost per outcome was related to the epidemiologic context (number of out-of-care individuals that each state was able to target for intervention), the service context (the total cost of each intervention), and the outcomes of each intervention (percent increase in proportion of patients virally suppressed). NC had by far the lowest cost per outcome ($920 per additional patient virally suppressed) as a result of being able to target 2,397 individuals for intervention; however, it had the least impact of the 5 interventions (47.5% increase in percent virally suppressed). VA and WI were able to target 303 and 308 individuals, respectively, and were associated with similar increased in the proportion of patients’ virally suppressed (54.6% and 58.4% increase in percent virally suppressed, respectively). However, VA cost less overall to implement and had, thus, lower cost per outcome ($2,212 versus $3,700 per additional patient virally suppressed, respectively). Similarly, LA and MA were able to target 147 and 105 patients and had the largest increases in the proportion of patients virally suppressed of our 5 interventions (91% and 78%, respectively). However, the LA intervention cost over twice as much to implement as the WI intervention. Therefore, the LA intervention had the highest cost per outcome of the 5 interventions ($4,415 per additional patient virally suppressed), and the MA intervention had the second lowest cost per outcome ($2,009 per additional patient virally suppressed). These results have the potential to inform the selection of appropriate settings and the design of future navigation-type interventions.

The role of surveillance data in demonstration state programs was of particular interest in this analysis. Findings from prior city- and state-sized studies support the use of surveillance data to identify PLWH most in need of targeted outreach and services and to promote efficient use of resources [37,38]. Surveillance data were found to be particularly helpful when states used them in planning. For example, when states made decisions about how to allocate funding based on surveillance data, programs were most efficient [29]. Our finding that programs that relied more heavily on surveillance data (MA and NC) had lower cost per outcome than the other 3 interventions adds an important nuance to growing body of surveillance data use literature.

Though results of this analysis indicate that public health costs associated with implementing patient navigation interventions to target newly identified or out-of-care PLWH are efficient, these findings do not come without limitations. First, because each of these programs was implemented within a state-wide system, it was not possible for us to identify appropriate internal comparators. Therefore, we were only able to compare data before and after implementation of the state-led navigation programs. However, we observed increases in the proportion of patients virally suppressed up to or near NHAS targets. In addition, the proportion of patients virally suppressed in this study was higher than in 9 of 11 HIV patient navigation interventions (49% to 71%) and much higher than usual care (30% to 39% percent) in a recent review of HIV patient navigation interventions [36]. Thus, it is likely that these interventions contribute to the outcomes we observed. However, for LA, it is likely that some patients would reengage in care without intervention after release from jail. The findings in this study are similar to or better than those observed in other jail settings with case management programs [35,36]. Therefore, it is likely the intervention in LA contributed to the observed outcomes. Second, these interventions were implemented in very different settings and targeted patients from disparate populations. We have employed inverse probability weighting to account for differences in patient populations across states and minimize bias in observed results that result from these differences. Third, it is not known whether these interventions would continue to be efficient if they were sustained over a long period of time and/or expanded across the entire state. If the programs studied in this analysis were to be scaled-up, our findings do not suggest one way or another whether, or at what point, saturation would be reached, or whether programs would achieve greater cost per outcome with scale. It is important to continue to follow linkage interventions in order to observe how cost behaves at different scales of operation.

## Conclusions

Patient navigation interventions and particularly those integrating the use of HIV surveillance data hold the potential to advance HIV care toward the goals of the NHAS [1], while maximizing cost per outcome within a context of limited resources. Previous cost-utility analyses found that interventions which targeted linkage and retention were either cost-efficient or cost-saving [18,39]. The incremental costs per patient and incremental cost per additional patient with viral suppression of the 5 demonstration state interventions fell below or within the range of previous research, and the additional number of patients virally suppressed fell above or within the range of previous research. In fact, the only 2 interventions in the previous cost-utility analysis that were at least as efficient as the 5 patient navigation intervention in this initiative (based on cost per additional patient with viral suppression) were judged as cost-saving. Thus, interventions in this initiative are likely to be at least as efficient as those in previous studies, if not cost-saving. The patient navigation-type interventions implemented in these 5 demonstration states substantially improved HIV care continuum targets over the first year of implementation. The cost per outcome of these interventions was better than or similar to previous patient navigation interventions. Patient navigation interventions that leveraged surveillance data to identify individuals who had never received HIV primary care or who were out of care were more efficient compared to interventions that used more traditional methods to identify out-of-care patients. Results showed that cost per outcome was maximized in settings where a high proportion of HIV–positive individuals had never received care or were out of care. Further research should focus on interventions that target HIV care continuum stages that experience the steepest drops-off, economies of scale, and the ideal contexts for patient navigation as an intervention strategy for both improved patient outcomes as well as cost-effectiveness.

## Supporting information

S1 CHEERS Checklist CHEERS, Consolidated Economic Evaluation Reporting Standards. (DOCX)

S1 Table Availability of data for analysis of viral suppression. (DOCX)

S2 Table Patient characteristics. (DOCX)

S1 Text Costing protocol overview. (PDF)

S2 Text Virginia Patient Navigation Protocol. (PDF)

S1 Data Costing template. (XLSX)

## Abbreviations

CDC: Center for Disease Control and Prevention
CI: confidence interval
DoH: Department of Health
ETAC: Evaluation and Technical Assistance Center
HRSA/SPNS: Health Resources and Services Administration’s Special Projects of National Significance Program
LA: Louisiana
MA: Massachusetts
NC: North Carolina
NHAS: National HIV/AIDS Strategy
PLWH: persons living with HIV
SLI: Systems Linkages Initiative
UCSF: University of California, San Francisco
VA: Virginia
WI: Wisconsin
