# Supplementary material for: Outcomes and costs of publicly funded patient navigation interventions to enhance HIV care continuum outcomes in the United States: A before-and-after study
Source: PLoS Med. 2021 May 13;18(5):e1003418. doi: 10.1371/journal.pmed.1003418 (PMC8118317; doi:10.1371/journal.pmed.1003418)
Supplement: S1 Table — (DOCX) [file pmed.1003418.s002.docx]

**S1 Table. Availability of Data for Analysis of Viral Suppression**

|  | **LA** | **MA** | **NC** | **VA** | **WI** |
| --- | --- | --- | --- | --- | --- |
| **Enrolled** | 147 | 151 | 2491 | 321 | 333 |
| **Viral load data missing at Baseline** | 0  (0%) | 10 (6.6%) | 319  (12.8%) | 39  (12.1%) | 14  (4.2%) |
| **Lost to follow-up before 12 months and did not achieve viral suppression** | 6  (4.1%) | 8  (5.3%) | 572  (23.0%) | 9  (2.8%) | 4  (1.2%) |
| **Final sample for assessment of viral suppression** | 141 (95.9%) | 133  (88.1%) | 1600  (64.2%) | 273  (85.0%) | 315  (94.6%) |
