# Supplementary material for: Outcomes and costs of publicly funded patient navigation interventions to enhance HIV care continuum outcomes in the United States: A before-and-after study
Source: PLoS Med. 2021 May 13;18(5):e1003418. doi: 10.1371/journal.pmed.1003418 (PMC8118317; doi:10.1371/journal.pmed.1003418)
Supplement: S2 Table — (DOCX) [file pmed.1003418.s003.docx]

**S2 Table. Patient Characteristics at Enrollment**

|  | **LA**  **N=147**  **n (%)** | **MA**  **N=151**  **n (%)** | **NC**  **N=2491**  **n (%)** | **VA**  **N=321**  **n (%)** | **WI**  **N=333**  **n (%)** |  |
| --- | --- | --- | --- | --- | --- | --- |
| **Demographic Characteristics** | | | | | | |
| Age – mean (stdev) | 42.3 (9.8) | missing | 40.3 (12.5) | 37.8 (12.6) | 35.5 (12.0) |  |
| Gender  Male  Female  Transgender | 123 (84%)  22 (15%)  2 (1%) | 99 (66%)  52 (34%)  0 (0%) | 1848 (74%)  637 (26%)  6 (<1%)* | 239 (74%)  82 (26%)  0 (0%) | 257 (77%)  69 (21%)  7 (2%) |  |
| Race/Ethnicity  African American  White  Latinx/Hispanic  Other/Mixed | 116 (79%)  26 (18%)  4 (3%)  1 (1%) | 52 (34%)  30 (20%)  66 (44%)  3 (2%) | 1782 (72%)  571 (23%)  110 (4%)  28 (1%) | 225 (70%)  59 (18%)  13 (4%)  24 (7%) | 205 (62%)  63 (19%)  57 (17%)  8 (2%) |  |
| Insurance Status  Private  Medicare  Medicaid  Other public  No insurance  Other/unknown insurance | 0 (0%)  0 (0%)  36 (24%)  0 (0%)  0 (0%)  111 (76%) | 4 (3%)  73 (48%)  5 (3%)  0 (0%)  3 (2%)  66 (44%) | 0 (0%)  0 (0%)  0 (0%)  0 (0%)  0 (0%)  2491 (100%) | 95 (30%)  94 (30%)  101 (31%)  5 (2%)  26 (8%)  0 (0%) | 42 (13%)  176 (53%)  104 (31%)  4 (1%)  6 (2%)  1 (<1%) |  |
| **Information about HIV Disease and Engagement in HIV Care** | | | | | | |
| HIV Risk Category  Heterosexual Sex  IDU  MSM  MSM and IDU  Other  Unknown | 20 (14%)  27 (18%)  36 (24%)  15 (10%)  0 (0%)  49 (33%) | 57 (38%)  39 (36%)  38 (25%)  6 (4%)  9 (6%)  2 (1%) | 372 (15%)  156 (6%)  1088 (44%)  70 (3%)  19 (1%)  786 (32%) | 106 (33%)  15 (5%)  169 (53%)  25 (8%)  6 (2%)  0 (0%) | 86 (26%)  29 (9%)  180 (54%)  18 (5%)  10 (3%)  10 (3%) |  |
| CD4 at Diagnosis – mean (stdev) | 390.9 (311.6) | 350.7 (278.3) | 392.6 (324.8) | 403.2 (310.3) | 399.2 (294.9) |  |
| HIV Care Engagement  Newly diagnosed  Never in care  Fallen out of care  At risk of falling out of care | 2 (1%)  0 (0%)  7 (5%)  138 (94%) | 36 (24%)  0 (0%)  72 (48%)  43 (28%) | 519 (21%)  55 (2%)  1389 (56%)  528 (21%) | 121 (38%)  20 (6%)  24 (7%)  156 (49%) | 149 (45%)  0 (0%)  50 (15%)  134 (40%) |  |

*2 participants were FTM Transgender.
