# Supplementary material for: Outcomes and costs of publicly funded patient navigation interventions to enhance HIV care continuum outcomes in the United States: A before-and-after study
Source: PLoS Med. 2021 May 13;18(5):e1003418. doi: 10.1371/journal.pmed.1003418 (PMC8118317; doi:10.1371/journal.pmed.1003418)
Supplement: S1 Text — (PDF) [file pmed.1003418.s004.pdf]

System Linkages

Economic Evaluation

Economic Evaluation

- Economic evaluation of this initiative will include two components:
  - Expenditure analysis
    - Global estimate of how grant resources have been expended
  - Costing
    - Estimate of real costs needed to replicate selected interventions

Economic Evaluation

- Analysis will focus on efficiency and may include:
  - Cost per HIV-infected patient identified
  - Cost per HIV-infected patient linked
  - Cost per HIV-infected patient retained
  - Cost per HIV-infected patient re-engaged
  - Cost per additional patient with undetectable viral load

Expenditure Analysis

- States will be asked to estimate how resources were expended by:
  - Intervention
    - Intervention 1, intervention 2, etc.
  - Expenditure category
    - Personnel, benefits, consultants, travel, etc.
  - Target
    - Identification, linkage, retention, re-engagement

Expenditure Analysis

- Expenditures associated with program management will be estimated separately:
  - Personnel, benefits, consultants, travel, etc.

Costing

- ETAC will work with demonstration sites to report the annual cost of implementation of demonstration site interventions.
- Costing categories include:
  - Personnel
  - Recurring costs
  - Capital investment (one-time costs)
  - Infrastructure (space)

Costing

- Demonstration sites will be asked to report on costs by:
  - Type of activity
    - Direct service, client contact; direct service, no client contact; indirect service
  - Intervention target
    - Identification, linkage, retention, re-engagement
  - Source of resources
    - HRSA SPNS grant vs. "in-kind" costs

Costing

- Costs associated with local and cross-site evaluation will be represented separately and are not included in the cost of demonstration site intervention implementation.

Next Steps

- Demonstration sites should identify one programmatic person (preferable one responsible for the budget) and one financial person to participate in economic analyses activities
- ETAC will develop and disseminate Excel templates to facilitate expenditure analysis and costing exercises

Next Steps

- ETAC will schedule follow-up calls with personnel identified for this activity to:
  - Review categorization of expenditures within state interventions
  - Finalize completion of templates
