# Supplementary material for: Outcomes and costs of publicly funded patient navigation interventions to enhance HIV care continuum outcomes in the United States: A before-and-after study
Source: PLoS Med. 2021 May 13;18(5):e1003418. doi: 10.1371/journal.pmed.1003418 (PMC8118317; doi:10.1371/journal.pmed.1003418)

Patient Navigation Strategy Protocol

Special Projects of National Significance (SPNS) Systems Linkages & Access to Care in Virginia

Patient Navigation Strategy Protocol
Special Projects of National Significance (SPNS)
Systems Linkages & Access to Care in Virginia

Protocol Contents:

Sections:		Page
	Introduction and Objectives	3
**I.**	Patient Navigation Referral Procedures and Coordination of Care and Services Agreement	4
**II.**	Client Intake Procedures	7
**III.**	Client Encounter Procedures	11
**IV.**	Client Transition Procedures	16
**V.**	Client Discharge Procedures	20
**VI.**	Procedures for Providing In-Home HIV Test Kits to Partners and Contacts	23

Appendices:

I. Coordination of Care and Services Agreement Form

II. Client Intake Package

III. Client Encounter Package

IV. Client Transition Package

V. Client Discharge Form

VI. Procedures for Providing In-Home HIV Test Kits to Partners and Contacts

VII. Service Categories for Patient Navigation Data Collection

VIII. Sample Patient Navigator Job Description

IX. Patient Navigator Training

X. Motivational Interviewing Summary

XI. Process Map for Making Active Referrals to Patient Navigation programs

XII. Process Map for Patient Navigation Intervention

Introduction:

Patient Navigation (PN) is an intervention that promotes linkage and retention in medical care through the guidance and support of health workers known as patient navigators. The aim of patient navigation is to help patients access the healthcare system so they receive the standard of care in a timely and effective way.[1] In Virginia, Patient Navigation is an intervention designed to link clients to care within 90 days of diagnosis and support retention in care for up to 12 months. PN also facilitates re-engagement in care.

Patient Navigators work with HIV positive clients to address/overcome barriers using Motivational Interviewing, provide client-centered education on how to access health and social service resources, and provide skills-training on how to interact with medical providers. Patient Navigators also link clients to necessary support services. The Patient Navigation intervention in Virginia also includes the facilitation of HIV testing by means of referrals and home test kits to identify and link positive clients directly to care and supports client transition into traditional services and self-management. A detailed process map highlighting Virginia's Patient Navigation intervention from referral to transition and discharge is located in the Appendices (Appendix XII: Process Map for Patient Navigation Intervention).

Patient Navigators received initial and on-going training on critical core competencies such as Motivational Interviewing, Fidelity Monitoring, client provider relationships, addressing barriers to care, field safety, cultural competency, linkage to care, disclosure and stigma, and other topics. The essential PN training components are outlined in Appendix IX: Patient Navigator Training. A sample Patient Navigator job description is located in the Appendices along with a summary of the Motivational Interviewing strategy.

The Patient Navigation Protocol was piloted in two clinical sites and one was replicated at a third site. Two sites were in rural areas and one was located in a major metropolitan center. The Protocol was implemented as described and presented in these sections.

Patient Navigation Objectives:

To support client linkage and retention in HIV care for up to 12 months by:

I. Facilitating entry into medical care for newly-diagnosed HIV-positive individuals, individuals who have been lost to HIV medical care, individuals "at risk" of dropping out of medical care, and individuals who have never entered the care system.

II. Providing client-centered counseling to assess and address client barriers to linking and retaining in HIV medical care.

III. Facilitating client linkage to medical care and needed support services and provide necessary follow up to support client retention in care.

IV. Providing client-centered education related to HIV disease, medical care, medications, and risk-reduction.

[1] *HIV Clinician*, Winter 2013, Vol. 25, No.1

V. Using Motivational Interviewing techniques to engage and empower client toward self-management.

VI. Transitioning clients to community services (such as case management) or self-management of their HIV medical care.

VII. Building relationships and communicating with providers and community agencies to accelerate and enhance client linkage and retention in care.

VIII. Facilitating with the early identification of individuals with HIV/AIDS by providing referrals to HIV testing for contacts and partners of clients and the distribution of in-home HIV test kits in circumstances where site referrals are not feasible.

Client Populations:

Patient Navigators will serve HIV-positive clients from the following client populations:

II. **Newly Diagnosed:** Individuals who have been diagnosed with HIV within the last 90 days.

III. **Lost to Care / Re-engaged**: Individuals who have not attended a medical appointment in 6 months or longer; or who have never been engaged in HIV care.

IV. **"At-Risk" of Falling out of Care:** Individuals who attend medical appointments sporadically and/or have other barriers that may affect their ability to stay in HIV care.

SECTION I:

Referral Procedures and Coordination of Care and Services Agreement:

SECTION I:
Referral Procedures and Coordination of Care and Services Agreement:

Procedure for Referring Patients to Patient Navigation Services:

Referral Agencies:
Patient Navigators actively recruit and receive referrals of HIV-positive clients from a variety of partners and agencies within the local system of care including, but not limited to:

- local health departments
- agencies that conduct HIV testing and referral services
- medical providers
- case managers

In some instances, a client is referred to Patient Navigation services by a Disease Intervention Specialist through the Virginia Department of Health SPNS Active Referral Protocol. The PNs work closely with DIS workers to ensure that clients are rapidly linked to HIV medical care upon a positive HIV diagnosis. A detailed process map for making active referrals to Patient Navigation programs is included in the Appendices (Appendix XI. Active Referrals to HIV Care Process Map).

Use of standardized Coordination of Care and Services Agreement Form**:**

1. Agencies that wish to refer a client to Patient Navigation services will use the standardized **Coordination of Care and Services Agreement** form.

   The purpose of this form is to gain informed consent from the client in order to coordinate their medical care, provide needed support services, and directly refer them to a Patient Navigator. The form allows the client to select which services they would like assistance with and the approved methods for contacting a client once the agreement has been signed.

   The agreement is valid for 24 months after the signature date. The form is designed for use by a variety of community agencies, local health departments, and testing and referral agencies. Please see ***Appendix I: Coordination of Care and Services Agreement*** form for the complete form and accompanying instructions.

2. **Sending a Client Referral to a Patient Navigation Program:**

   The referring agency should complete **page 1** of the **Coordination of Care and Services Agreement** form and send it to the Patient Navigation program, including client signature. Referral forms can be received via secure fax, through a secure electronic database or by secured email system (scanned signed copy), or an initial verbal referral by phone if applicable. If the referral is received by phone, written client consent must be still obtained on the form and provided to the Patient Navigation program.

Procedure for Patient Navigation Program Receiving Client Referrals:

1. **Navigator/Client Assignment process:** Patient Navigator assignment is conducted by the local Patient Navigation program or agency on the basis of Patient Navigation caseload (acuity and number of clients) or based on geographic location of client and/or Patient Navigator.

2. **Receiving and Processing Referrals:** From the date that the referral was received by the Patient Navigation program, the assigned Patient Navigator should contact the client within 72 hours to schedule or confirm the client's medical appointment and schedule an in-person intake appointment for Patient Navigation services.

    i. **Methods of Contact:** The Patient Navigator should utilize the client-approved methods of contact indicated on the **Coordination of Care and Services Agreement** form only (i.e. cell phone, text messages, email, etc.). If text messaging or email is used, the PNs should follow their agency's client electronic communication policy so that no breach in confidentiality occurs.

    ii. **Contact Attempts:** Patient Navigators should make **at least three** attempts at different times to contact the client through the approved methods of contact within one week from receipt of referral.

    If the client has authorized more than one method of contact (phone, in-person, text message, email), the Patient Navigator should attempt at least one other method of contact that is different from previous attempts:

    EXAMPLE:
    Attempt 1 within 72 hours of referral: Cell phone/voicemail
    Attempt 2 within 5 days of referral: Text Message
    Attempt 3 within 7 days of referral: In-Person or another phone number

    iii. **Scheduling Patient Navigation Intake Visit**: Once client contact has been established, the Patient Navigator should schedule a Patient Navigation intake visit to meet with the client in-person. If possible, the Patient Navigator should also schedule or confirm their first medical appointment during this initial client contact.

        a. If the Patient Navigator is able to schedule or confirm the client's first medical appointment during this initial contact, the Patient Navigator should complete **page 2** of the **Coordination of Care and Services Agreement** form and provide it to the referring agency/individual. Ideally, the PN and client should make the call together or the client should make the call with the PN present.

b. The Patient Navigator should confirm the client's ability to transport his or herself to the intake visit and offer support services (i.e., bus tokens or taxi vouchers) or a field visit if necessary.

SECTION II:

Patient Navigation Client Intake Procedures

SECTION II: Patient Navigation Client Intake Procedures

Patient navigators will use **Appendix II: Client Intake Package** to document the client intake visit including the Client Intake Visit Checklist, the Client Contact Information Sheet, the Client Assessment Form, and the Client Linkage to Care Plan (LCP). It is recommended that the PN use Motivational Interviewing techniques to develop a rapport with the client before completing the forms. The PN is encouraged to make the client more relaxed and at ease by being friendly and welcoming which will accelerate rapport and reduce the client's anxiety. Ice breaking examples include asking questions such as "How are you doing today? "Where were you born? "How was your weekend?" that will put clients at ease and make them more receptive to Patient Navigation services.

Client Intake Visit Checklist:

The ***Client Intake Visit Checklist*** contains the necessary elements that should be completed at a client intake visit. The Patient Navigator should fill this checklist out as they complete components of the intake visit. The Patient Navigator should also document the start and end time of the client intake visit on the checklist. The checklist represents the core/minimum activities that should occur at a client intake visit. Additional activities or issues addressed should be documented in the notes section of this form. This document should be stored as the first page of the Client Intake Package in the client's file.

- ✓ Complete **Client Contact Information Form**
- ✓ Conduct necessary Financial Screening for Services Eligibility (Ryan White and other)
- ✓ Facilitate or confirm next client medical appointment and document date of appointment
- ✓ Fax back confirmed date of medical appointment to referring agency using page 2 of the **Coordination of Care and Services Agreement Form**
- ✓ Conduct Initial Barriers Assessment using the **Client Assessment Form** and complete a **Linkage to Care Plan** (LCP)
- ✓ Make a copy of the LCP for the client to take home (unless client refuses copy) – do not include Client ID on the client's copy of their LCP
- ✓ Facilitate referrals to HIV testing for client contacts or partners (if applicable in encounter setting)
- ✓ Provide tailored client education and document components covered in client's record
- ✓ Complete the **Client Encounter Form** (ensure that outcome and process data is recorded in electronic database)

1. Client Contact Information Form

i. The ***Client Contact Information Form*** should be filled out first at the client intake visit and should be updated at each subsequent client visit. The purpose of this form is to provide the Patient Navigator with the most up-to-date client contact information including address, phone, email address and the best way to contact the client between visits. Clients should also be asked if they would like to provide contact information for

up to two alternate contacts and whether these individuals can be contacted on behalf of the client. Clients are not required to provide contact information for any alternate contacts.

ii. It is important to update or confirm information on this form at each client visit to ensure that the Patient Navigation program has accurate information on transient clients or clients with unstable or sporadic housing situations. If the client wishes to provide a different address or contact method other than their permanent address, this form can be used to capture that information to maximize the Patient Navigator's ability to communicate with the client.

2. Screening and Eligibility of Services

The client should be screened for eligibility of services including Ryan White and Medicaid at the client intake visit. If the Patient Navigation program works with a provider or case manager to conduct this process, this should be noted on the Intake Visit Checklist. The Patient Navigation program should utilize established practices within their agency for assessing eligibility.

3. Facilitate or confirm medical appointment

i. **Verifying Services Eligibility:** Prior to scheduling a medical appointment with an HIV provider, the eligibility intake process should be complete so the client is enrolled in Ryan White or other benefit assistance program. Once eligibility is complete, the client is informed of the available options for HIV care in the area (private providers, Ryan White funded clinics, etc). During this process, the client's financial responsibility for care (if any) should be discussed so that the client can make an informed decision. Referrals to the Virginia AIDS Drug Assistance Program (ADAP) should follow established ADAP protocols.

ii. **Scheduling and verifying medical appointments:** The first medical appointment should take place **within 2 weeks** (if possible) of the Patient Navigator establishing contact with client. The Patient Navigator should work with the client to make the appointment following the established scheduling procedures at the clinic site. If the client already has an appointment, the Patient Navigator should record this information in the client's paper chart and/or in the electronic database.

iii. Upon scheduling or verifying the client's first medical appointment **(within 2 weeks of initial client contact)**, the Patient Navigator should complete **page 2** of the **Coordination of Care and Services Agreement** form and fax it back to the referring agency/individual to confirm linkage to care.

iv. The Patient Navigator should also verify attendance at the medical appointment with the referring agency using **page 2** of the **Coordination of Care and Services agreement within 30 days** of initial client contact unless there are extenuating circumstances that prevent the client from attending an appointment within this timeframe.

4. Conduct initial Barriers Assessment and complete Linkage to Care Plan (LCP)

i. Using the **Client Assessment Form** and the **Linkage to Care Plan (LCP)** as resources, the Patient Navigator should use client-centered Motivational Interviewing skills to explore potential barriers for linking into medical care, with a particular focus on barriers to attending their first or next medical appointment.

ii. The Patient Navigator should record notes related to these barriers on the LCP document and explore possible solutions to overcome these barriers with the client. These plans should be documented by the Patient Navigator in the Solution/Plan to Overcome Barrier section of the form and include client-driven target dates for resolving these barriers.

iii. The Patient Navigator **should offer the client a copy of the LCP (without the client identifier on it)** at the end of the visit. If the client refuses a copy of the LCP, this should be noted on the checklist. At each subsequent visit, the Patient Navigator will conduct another barriers review and assessment using the Retention in Care Plan (RCP).

5. Offer client partner/contact HIV testing services

See Section VI. Procedures for Providing Home HIV Tests

6. Provide tailored client-centered support and education

At each client encounter (including intake), the Patient Navigator should use Motivational Interviewing techniques to engage the client in discussion on one or more of the following topics:

- Basic individualized HIV education (HIV viral life cycle, lab values, medications, etc.)
- HIV medical care basics and what the client should expect during medical appointments
- Safe sex practices
- HIV disclosure issues
- Treatment adherence support
- Substance use and harm reduction
- Other individualized topics specific to the client's needs

7. Conduct necessary data/reporting for intake visit

Patient Navigators should capture all needed data elements for a client intake visit in their electronic database management system or via the provided data reporting forms.

SECTION III:

Patient Navigation Client Encounter Procedures

SECTION III: Patient Navigation Client Encounter Procedures

Patient navigators will use **Appendix III: Client Encounter Package** to document client encounters including the Client Visit Checklist, the Client Contact Information Sheet, and the Retention in Care Plan (RCP). The types of active PN encounters that are recommended are by telephone, in the office, in the field (including doctor's office), or at the client's home.

Client Encounter Check-List:
The ***Client Encounter Checklist*** contains the necessary elements to be completed at every client visit (other than the intake visit). The Patient Navigator should fill this checklist out as they complete components of the visit. The Patient Navigator should also document the start and end time of the client visit on the checklist. The checklist represents the core/minimum activities that should occur at every client visit. Additional activities or issues addressed should be documented in the notes section of this form. This document should be stored in the client's file.

- ✓ Update **Client Contact Form**
- ✓ Facilitate or confirm next client medical appointment and document date of appointment
- ✓ Review client accomplishments and ongoing barriers and complete the **Retention In Care Plan** (RCP) form
- ✓ Make a copy of the RCP for the client to take home (unless client refuses copy) – do not include Client ID on the client's copy of their RCP
- ✓ Make referrals to support services to address barriers listed in RCP if applicable
- ✓ Facilitate referrals to HIV testing for client contacts or partners if applicable
- ✓ Provide tailored client education and counseling and document components covered in client's record
- ✓ Complete the **Client Encounter Form** (ensure that outcome and process data is recorded in electronic database)

1. Update Client Contact Form

At each client encounter/visit, the Patient Navigator should update the client's contact information using this form. This is an opportunity for clients to share new addresses, phone numbers, and alternate contact information with their Patient Navigator. Clients are not required to provide alternate contacts on this form.

2. Facilitate or confirm medical appointment

The Patient Navigator and client should make the appointment following the established scheduling procedures at the clinic site. If the client already has an appointment, the Patient Navigator should record this information in the client's paper chart and/or in the electronic database.

3. Complete Retention in Care Plan (RCP)

At each client encounter or visit, the Patient Navigator will use client-centered approaches to engage the client in identifying and addressing barriers to staying in HIV care. The top portion of the Retention in Care Plan (RCP) is designed to help the client and Patient Navigator review accomplishments and barriers that have been overcome since the last encounter as well as ongoing barriers that may persist. The form is designed to facilitate a client-driven discussion identifying new barriers and ways to overcome them. The Patient Navigator should use client-centered Motivational Interviewing techniques to facilitate discussion with the client.

The Patient Navigator should document notes on barriers and the plan to overcome them on the RCP document. Like the Linkage to Care Plan, a copy of the RCP should be given to the client at the conclusion of the visit (without client name or ID). If the client refuses a copy of his/her RCP, make a note on the Encounter Checklist.

4. Make Referrals to Supportive Services

i. **Assessment for determining need for support services**
The need for support services is assessed from the review of LCPs and RCPs. Support services needed are then documented in the client file and/or electronic database system and referrals made and confirmed accordingly.

ii. **Process for referring to support services**
The process should follow established referral procedures at the site. If an electronic system is in place, referrals should occur accordingly. If there is no electronic system, paper copies should follow established guidelines of the site receiving the referral. Regardless of the method, verification and documentation processes should be followed.

5. Facilitate referrals for partner/contact HIV testing

See Section VII. Procedures for Providing Referrals and In-Home HIV Tests

6. Provide tailored client education/support/counseling

At each client encounter, the Patient Navigator should use Motivational Interviewing techniques to engage the client in discussion on one or more of the following topics:

- Basic individualized HIV education (HIV viral life cycle, lab values, medications, etc.)
- HIV medical care basics and what the client should expect during medical appointments
- Safe sex practices
- HIV disclosure issues
- Treatment adherence support
- Substance use and harm reduction
- Other individualized topics specific to the client’s needs/requests

7. Document Client Encounter

After each client encounter/visit, the Patient Navigator should document relevant data from the visit in the site's electronic reporting system. Refer to Appendix VII: Service Categories for Patient Navigation Data Collection for specific service category required options to select based on the type and outcome of the encounter/visit.

8. Client Follow-Up and Engagement Procedures

Client Engagement: Client engagement in care should attempt to overcome a variety of factors that the client may be dealing with, including but not limited to: homelessness, substance abuse, stigma, poverty, lack of transportation, fear/ mistrust of the medical system and impatience with red tape and bureaucratic hassles. To achieve this, the Patient Navigator should actively assess LCP and RCP for trends and progress towards goals. If goals are not being met, an assessment should occur to determine new or continuing barriers.

Engaged Clients: Clients who attend HIV medical appointments as scheduled and take their HIV medications as directed.

Frequency and type of contact:

- **First 90 days (Months 1-3 of client engagement):** Client contact should be more intensive, face to face contact, with a focus on barriers to linkage and retention in care. The LCP and the RCP should be reviewed during each client contact. Phone, text or email reminders of medical and support service appointments should be made by the Navigator during this time. Support services such as transportation assistance and/or accompaniment to appointments, should be provided as needed.

- **Subsequent 6 to 9 months (Months 4-12 of client engagement):** Contact during this timeframe may be decreased for engaged clients. However, barriers to retention in care should still be addressed. Contacts may be made by phone, text, email, and/or in-person. Both the LCP and RCP should be reviewed for progression to transition every 6 months.

Non-Engaged Clients: Clients who do not keep medical appointments as scheduled and/or do not take their HIV medications as directed.

Frequency and type of contact:

First 90 days (Months 1-3 of client engagement): Intensive, face-to- face contact, focused on barriers. The LCP and the RCP should be reviewed during each client contact. Client phone, text, email reminders should be made by the PN for medical and support service appointments during this time. Support services such as transportation assistance and/or accompaniment to appointments, should be offered as needed.

- **Subsequent 3 to 6 months (Months 4-6 of client engagement):** Contacts may be phone, text, email, and/or in-person. The LCP and the RCP should be reviewed during each client contact. Encounters should focus specifically on adherence to medication and attendance at medical appointments. The Patient Navigator should explore the need for additional support services and may continue to transport and/or accompany the client to appointments.

- **Subsequent 6-9 months (Months 9-12 of client engagement):** Contacts may be phone, text, email, and/or in-person. The LCP and the RCP should be reviewed for progression to transition. Encounters should focus specifically on adherence to medication, attendance at medical appointments, and moving toward self- management practices. The Patient Navigator should explore the need for additional support services and may continue to transport and/or accompany the client to appointments.

Missed Medical Appointments:
Procedures for following up on missed medical appointments should follow established procedures at the clinic site. If no procedure is in place, the Patient Navigator should contact the client to review barriers to attending medical appointments and offer support services to assist with transportation, housing, and other assistance as needed. Attempts and results should be documented in the client's file and electronic database.

SECTION IV:

Patient Navigation Client Transition Procedures

SECTION IV: Patient Navigation Client Transition Procedures

From the beginning, the Patient Navigator and client should have a clear understanding of what should occur before patient navigation services ends. The process of discharge and transition planning should begin at the time of intake when the client agrees to become a Patient Navigation client. Transition is the process of moving from one level of care to another, or leaving care.[2] Discharge is defined as the point at which an individual's active involvement with services is terminated, and the client is no longer a client of the program.[3]

The client should be engaged in all levels of transition and discharge planning. The client should be continuously reassessed for transition readiness based on a set of criteria established by the client and Patient Navigator. Successful transitional planning should consider a client's medical, physical, cognitive, economic and emotional strengths and abilities as well as their available support systems.

See Appendix IV: Client Transition Package to document client transition on the Client Assessment form and Client Transition Plan.

Assessing Client for Transition from Patient Navigation Services:

1. Each client should be assessed for readiness to **transition/discharge at least every 6 months**. For Ryan White clients, it is recommended that, if possible, this form be completed while conducting Ryan White recertification, which also occurs every 6 months.

2. The Patient Navigator should use the **Client Assessment Form** to assess the client's readiness for transition.

3. The Patient Navigator should explain the purpose of the form to the client. The purpose of the form is to review the client's barriers to HIV medical care every six months in order to assess the client's readiness to transition from Patient Navigation services.

4. If the client identifies all of the same barriers prior to transition or discharge as they did at the time of intake, further assessment is needed to determine if the client is appropriate for transition and discharge from Patient Navigation services.

5. When using this form to assess client readiness to transition from Patient Navigation services, the Patient Navigator should fill out Section 3 with the date of the client's most recent HIV medical appointment and the date of the client's most recent HIV medication pick-up.

[2, 3] *Planning for Transition and Discharge, Part 1*. Retrieved from http://www.lanstat.com/newsletter/transition1.html.

6. If it is determined that the client is not ready for transition from Patient Navigation services due to continuing barriers to staying in care or sustaining HIV medications, the client can remain in PN services until re-assessed within a 6-month time period. The reason for the decision to transition or not to transition a client from PN services must be documented on the form.

7. Original copies of the completed forms should be kept with the client's Patient Navigation care plan files.

8. The Patient Navigator should also document data from the **Client Assessment Form** in the agency's electronic data system (or the state Ryan White system) along with information from the client's **Linkage to Care Plan, Retention in Care Plan,** and **Client Transition Plan**.

Completing the Client Transition Plan:

When a client has been determined eligible for transition or discharge from Patient Navigation services, the Patient Navigator will work with the client to complete the Client Transition Plan. Clients should be assessed for transition/discharge from Patient Navigation services at least every six months.

The following should be considered during the transition assessment process between the Patient Navigator and client:

- Attendance at HIV medical care appointments (2 or more medical appointments within 6 months)
- Verification of medication pick-up
- An assessment of barriers and needs documented on the Client Assessment form and Client Linkage and Retention in Care Plans

The following information must be provided in the Client Transition Plan:

1. **Client information:**
    a. Transition Plan date (today's date)
    b. Client ID (agency-specific)
    c. Patient Navigator name
    d. Date of client's most recent HIV medical appointment
    e. Date of client's most recent HIV medication pick-up

2. **Transition Service Information:**
   Indicate the services that the client will transition to after discharge from Patient Navigation services. List the name of the agency and provider (if applicable). More than one box may be checked if the client will be working with both a medical and non-medical case manager after discharge.

3. **Summary of Resolved and Unresolved Needs/Barriers:**
   The Patient Navigator should use client-centered Motivational Interviewing techniques to facilitate discussion with the client:
   a. For each "Indicated Need/Barrier", summarize the issues that have been resolved during the client's enrollment in Patient Navigation services in the "Resolved" column.
   b. For each "Indicated Need/Barrier", summarize the issues that still require action after the client's discharge from Patient Navigation in the "Unresolved" column.
   c. Enter the individual responsible for taking action on any unresolved issues in the "Responsibility" column.

4. **Transition Plan Agreement:**
   The client, Patient Navigator, and Patient Navigator supervisor should sign the form to indicate approval of Transition Plan prior to discharge from Patient Navigation services. If the client will be transitioned to case management or other services, the case manager or other transition personnel should review and sign this plan with the client and Patient Navigator present. If it is not possible to have the case manager or transition personnel present, the Patient Navigator should send this form to the case manager or transition personnel for review. The case manager or transition personnel should sign the form and send it back to the Patient Navigator.

SECTION V:

Patient Navigation Client Discharge Procedures

SECTION V:
Patient Navigation Client Discharge Procedures

1. The Patient Navigator will use the Client Discharge Form (Appendix V) to document discharge from Patient Navigation services.

Patient Navigation Discharge Information:

a. Patient Navigator Name: In addition to documenting the Client ID at the top of the form, this section should include the **name of the Patient Navigator** that will be transitioning the client and;

b. Date of Discharge from Patient Navigation services

c. Discharge Reason: The Patient Navigator should indicate why the client is being discharged from Patient Navigation services in this section. Reasons for discharge are listed below.

- Service Completion: The client has completed services with the Patient Navigation program and has been transitioned out of services.
- Unable to Contact: The Patient Navigator has been unable to contact the client for 3 or more months.
- Patient Navigator request: The Patient Navigator requests to discharge the client or to transfer the client to another Patient Navigator.
- Client request: The client requests to be discharged from Patient Navigation services.
- Transferred to another Patient Navigator: The client is transferred to another Patient Navigator. In this case, the Patient navigator should indicate where and who.
- Moved out of Service Area: The client has moved out of the service area of the Patient Navigator. If possible, document where the client has moved.
- Deceased: The client is deceased.
- Incarcerated: The client has become incarcerated.
- Other: Document other reasons for discharge.

d. Discharge Service Level: The Patient Navigator should document how the client has been transitioned and discharged from Patient Navigation services:

- Self-Management: The client will be discharged and will self-manage their retention in care.
- Non-Medical Case Management: The client will be discharged and has been assigned a non-medical case manager. Note: the Non-Medical Case Manager should review the Transition Plan and client barriers as stated in the Transition protocol.

- Medical Case Management: The client will be discharged and has been assigned a medical case manager. Note: the Medical Case Manager should review the Transition Plan and client barriers as stated in the Transition Plan.
- Other Patient Navigator or Community Health Worker: The Patient will be discharged from services but may be working with another Patient Navigation program to coordinate services.
- Undetermined/Unknown
- Other: Please document other services that the client may be discharged to.

e. If applicable, document the name of the agency providing case management or other services in this section of the form.

f. Date of Most Recent Medical Visit: Document the date of the client's most recent medical visit.

g. Date of Most Recent HIV Medication Pick-Up: Document the date of the client's most recent HIV medication pick-up.

h. Document the client's housing status into one of the four categories:
- Stable/Permanent
- Unstable
- Temporary
- Unknown

i. Document the client's current insurance status and select all that apply:
- Private
- Medicare
- Medicaid
- No Insurance
- Unknown
- Other: Specify type if known.

j. Client Discharge Approval: Both the Patient Navigator and the Patient Navigator Supervisor should review and sign off on the discharge of the client from Patient Navigation services. Agencies are free to establish their own review procedures; however it is necessary to have a supervisor or designated staff person review and approve client discharges.

k. Notify client of discharge from Patient Navigation services:
- Depending on the nature of the client transition and discharge, the Patient Navigator should make at least three attempts to contact the client to notify them of discharge from services.

SECTION VI:

Procedures for Providing In-Home HIV Test Kits to Partners and Contacts

SECTION VI:
Procedures for Providing In-Home HIV Test Kits to Partners and Contacts

Virginia Department of Health Division of Disease Prevention
Guidelines for Providing In-Home OraQuick® Tests

The Virginia Department of Health (VDH) prefers that people receive HIV testing in a setting that can provide appropriate pre and post-test counseling, answer questions, and link the patient to care and support services as needed. There are times, however, when individuals are unable or unwilling to present for testing. The in-home test provides additional testing options that Patient Navigators can distribute to individuals in the field or at a home visit for ensuring that individuals are tested and learn their HIV status. The in-home tests are only to be used for individuals who are 17 and older.

As appropriate, counselors should first encourage people to conduct an HIV test on-site. Individuals who are offered in-home tests should be high-risk individuals who are unable or unwilling to get tested through an established medical, community, or outreach location. In-home tests may also be provided to individuals who identify others in their social networks who are at risk for HIV, but unwilling to test on-site or have challenges related to transportation/distance from a test site.

Ideally, test reporting information shall be requested for each in-home test. Each testing site needs to report the number of tests distributed and, if possible, obtain basic demographic information (race, gender, age, risk if known) and test results. The collection of this information may occur in a number of ways (online, by phone, in person). See the attachment for a template data collection form that may be individualized by agency.

Testing sites need to ensure that in-home test kit recipients have contact information for counseling and follow up testing. While each kit already contains a national hotline number set up by OraSure Technologies, Inc, participating agencies must also include additional referral information, such as the Virginia Department of Health Hotline, the agency contact number, and instructions for next steps based on the test result.

Sites should seek a commitment from people to actually use the test, whether or not they report back to the agency, as the tests are expensive. Clients who receive test kits for distribution should be advised that the test kits may not be sold and cannot be returned to drug stores for cash as the boxes are not coded for individual sale. Agencies should also set an initial limit on the number of test kits that individuals may receive.

Clients should be counseled that, if they plan to use the test to verify the status of sex partners prior to engaging in sex, they need to be aware that:

- the test may not pick up infection that occurred within the last three months;

- they should not use a negative test result as an indicator that it is safe to engage in unprotected sex; and they need to consider the implications of a sex partner learning their HIV status in the client's home or location where they are engaging in sex (e.g., reaction to a test result, need for additional assistance, etc).

Innovative approaches, such as using Skype or i-Chat to provide pre and post counseling, should be discussed and approved by the DDP contract monitor in advance. Testing sites interested in providing an incentive for the in-home test should contact their contract monitor for guidance. A brief survey will be distributed by DDP to contractors and clients to assess this new testing method.

SPNS Patient Navigation Guidance for use of In-Home OraQuick® Tests

Role of Patient Navigators:

Patient Navigators are uniquely positioned in community health settings to assist with the early identification of individuals with HIV/AIDS because they have developed important relationships and connections with their patients and communities. By equipping Patient Navigators with in-home test kits, VDH hopes to expand the reach of HIV testing efforts and identify HIV-positive individuals in hard-to-reach populations. Furthermore, Patient Navigators can facilitate rapid linkage to HIV care services for clients that test positive.

When to Use In-Home Tests:

- The SPNS Patient Navigation protocol emphasizes the important role the Patient Navigator plays in the early identification of individuals with HIV/AIDS. Therefore, in applicable situations, such as field or home visits, the Patient Navigator should discuss the availability of testing services for partners, contacts and family members at community testing sites.
- After discussing the benefits of taking an HIV test and receiving the results in a setting that can support necessary pre and post-test counseling and linkage to care services, if an individual refuses to be referred or accompanied to an HIV test site, an in-home test may be provided by the Patient Navigator.
- SPNS Patient Navigators are provided with one box of OraQuick® In- Home tests (6 tests total) for use the above-mentioned situations at a time. One of the six tests should be repackaged into the Patient Navigator's SAMPLE TEST KIT and clearly labeled accordingly. The SAMPLE TEST KIT can be used to demonstrate and familiarize clients and partners with administration of the test.

Administration of In-Home Tests:

- Depending on the situation, the Patient Navigator may or may not be present during the administration of the in-home test:
- If the Patient Navigator is present during the administration of the in-home test, VDH recommends that the Patient Navigator provide a brief demonstration of how the test is self-administered using their sample test kit. However, the client should self-administer the test without direct assistance from the Patient Navigator.
- If the Patient Navigator is providing an in-home test to a partner/contact or other third party and is not present during the administration of the test, VDH recommends that the Patient Navigator provide a brief demonstration to the contact or third-party distributer of how the test is self-administered using their sample test kit.
- Please note that The OraQuick *ADVANCE*® Rapid HIV-1/2 Antibody Test should never be stored at temperatures exceeding 80°F.

Follow-Up Resources:

The Guidelines require that sites using in-home test kits ensure that test-kit recipients have contact information for counseling and follow-up testing services, the HIV/STI hotline, the PN agency contact number and instructions for the next steps based on the test results. In addition, SPNS Patient Navigators are also required to include a business or contact card with their individual contact information.

Documenting Distribution of Test Kits:

SPNS sites should capture information on the distribution of In-Home test kits using the data collection log provided by VDH. (See attached template). SPNS sites should submit this log to VDH on a monthly basis with their contractor monthly reports. If no in-home tests were distributed by the site within that month, this should be indicated on the monthly contractor report.

Sites may opt to use the supplemental data collection insert document attached and/or use a survey monkey to collect information from individuals using in-home test kits.

Patient Navigation Strategy Protocol Appendices

Special Projects of National Significance (SPNS) Systems Linkages & Access to Care in Virginia

Appendices:

I. Coordination of Care and Services Agreement Form

II. Client Intake Package

III. Client Encounter Package

IV. Client Transition Package

V. Client Discharge Form

VI. Procedures for Providing In-Home HIV Test Kits to Partners and Contacts

VII. Service Categories for Patient Navigation Data Collection

VIII. Sample Patient Navigator Job Description

IX. Patient Navigation Training

X. Motivational Interviewing Summary

XI. Process Map for Making Active Referrals to Patient Navigation programs

XII. Process Map for Patient Navigation Intervention

Appendix I.

Coordination of Care and Services Agreement Form

Place Agency Name Here:____

Coordination of Care and Services Agreement Form
Instructions

Purpose of Form:
The purpose of this form is to allow the client and the agency that provides services to identify and select available community resources. The goals are to help coordinate services, assist with closing the referral loop, and allow for easier linkages to care.

Each agency that initiates this form becomes the owner of this form and their agency name should be placed at the top of the page. Each agency should decide if this form will replace or supplement their current consent for services and or release of information form(s). Each agency will also need to decide how and where they want to store and maintain this form.

The client will then have the opportunity to agree to services in a program(s), that may provide a continuum of care and services with distinctive agency responsibilities and to ensure seamless service delivery. It is presumed that when an agency is checked by a client on this form to request services, the Agency will initiate the first contact with the client ***unless specified in writing that the client will make first contact.***

The instructions on this page will help with the completion of the Coordination of Care and Services Agreement Form.
Please remember, if the form is not complete and accurate, this may cause a delay in obtaining additional services for the client.

Instructions for Form Page 1:

1. The agency representative will print the client's full name, address and date of birth on the top portion of the form.

2a. Check the appropriate box to indicate the client's medical diagnosis (HIV/AIDS and/or Hepatitis C) and write in the corresponding diagnosis date for each diagnosis.
2b. Check the appropriate box for current gender of the client.
2c. Check the appropriate box indicating the client's race.
2d. Check the appropriate box indicating the client's ethnicity.

3a. Check the appropriate confidential information that the client wishes to exchange, writing in any additional information not listed.
3b. Check the appropriate box indicating that the information listed in 3a can be released to help assist with the listed care arrangements and/or providers as specified on page 2 of this form. This form is not intended to be a blanket consent form and information should only be exchanged with the agencies listed on page 2 of this form.

4. The client will need to advise agency representative the best contact method(s) and if it is appropriate to leave a message on the phone or at work.

5. The agency representative will write in the authorization effective date and advise the client that the authorization date is valid for 24 months from the signature date. If revoked, the **client must sign and date**. The client is responsible for contacting the agencies to withdrawal from their services.

6. The client will sign and date the form acknowledging the purpose of the form.

7. The agency representative will complete their name, address and phone number.

Instructions for Form Page 2:

Section A: This section should be filled out by the agency representative who originated the form.
1a. Print the client's full name.
1b. Print the client's date of birth.
1c. List the name of the organization(s) that the client is being referred to for medical care or other services.
1d.The agency representative who originated the form should print their own name, secure fax line, and phone number.

Section B: This section should be filled out by the agency representative who received this form and who will be coordinating care for the client.
2. The Patient Navigator or other Linkage Personnel will write in their name, agency, phone number, and secure fax line.

3. The Patient Navigator or other Linkage Personnel using this form will complete information related to the referral for medical care including the name of the agency or provider that the client is being linked to, the date of the referral, and the date of the medical appointment.

4. Once attendance to the **medical appointment is confirmed**, the Patient Navigator or Linkage Personnel at the provider site will confirm the date of attendance and circle how the original agency was notified of attended appointment. If sending confirmation by fax, **please be sure to use a fax cover sheet.**

5. Referrals to other services can be recorded in the subsequent lines provided on page 2 of the form.

Place Agency Name Here:____

COORDINATION OF CARE AND SERVICES AGREEMENT

I understand that different agencies provide different services and benefits, and that each agency must have specific information to provide services and benefits. By signing this form, I allow agencies to use and exchange certain information about me so it will be easier for them to work together efficiently to provide or coordinate these services or benefits.

1) I, ____am signing this form for the opportunity to receive coordination of services.
(Print Client's Full Name)

____ ________________________

Client's address **Client's Date of Birth**

2a) Client Diagnosis:

- ☐ HIV/AIDS — HIV/AIDS Diagnosis Date:_______________________
- ☐ Hepatitis C (HCV) — Hepatitis C (HCV) Diagnosis Date:_________________

2b) Current Gender of Client

- ☐ Male
- ☐ Female
- ☐ Transgender – M2F
- ☐ Transgender – F2M
- ☐ Transgender – Unspecified
- ☐ Declined
- ☐ Other, Specify:_________

2c) Client Race:

- ☐ American Ind./AK Native
- ☐ Asian
- ☐ Black/African American
- ☐ Native HI/Pac. Islander
- ☐ White
- ☐ Don't Know
- ☐ Declined

2d) Client Ethnicity:

- ☐ Hispanic or Latino
- ☐ Not Hispanic or Latino
- ☐ Don't Know
- ☐ Declined

3a) I allow the following confidential information about me to be shared (check all that apply):

- ☐ Contact information
- ☐ Medical Diagnoses
- ☐ Mental Health Diagnosis/Treatment
- ☐ Demographic Information
- ☐ Medical Appointments
- ☐ Substance Use Diagnosis/Treatment
- ☐ Financial Information
- ☐ Individual Services Plan
- ☐ Client's testing information
- ☐ Other:________________

Information in the boxes indicated above may be updated and shared with the providers indicated in **3b.** **Yes** ☐ **No** ☐

3b) I consent that the information indicated in item 3a can be released for the following care arrangements (check all that apply) <u>as specified on page 2 of this form</u>:

- ☐ Medical Care Providers
- ☐ Other Core Medical Services
- ☐ Mental Health/Substance Use Services
- ☐ Medication Access
- ☐ Other Support Services

4) I may be contacted by the following methods (check all that apply):

- ☐ In person only, at this location: ____
- ☐ Postal Mail/Letter. Address, if different from above: ____
- ☐ Home Phone: ___________________ May we leave a message? (circle) YES NO
- ☐ Cell Phone: ___________________ May we leave a message/text message? (circle) YES NO
- ☐ Work Phone: ____________________ May we leave a message? (circle) YES NO
- ☐ Email: _________________________

5a) This agreement is effective: ________________
(Date of Agreement)

5b) If revoked, check box, sign and date ☐ ________________________
(Sign and Date if revoking agreement)

It is understood that this agreement for the **<u>coordination of my care services</u>** is valid for **<u>24 months</u>** from the agreement date.

In addition, it is understood that in order to assist in the coordination of my care, a health system navigator (HSN), or patient navigator (PN), or other type of linkage to care staff or personnel can attempt to contact me by the above-approved methods, in the event that I miss a scheduled medical or other type of appointment related to my HIV care.

I can withdraw this agreement at any time by informing all referred agencies. The listed agencies must stop sharing information after I inform them that my authorization has been withdrawn. I have the right to know what information about me has been shared, why, when, and with whom it was shared. If I ask, each agency will show me this information. I want all agencies specified to accept a copy of this form as valid consent to share information. If I do not sign this form, information will not be shared and I will have to contact each agency individually to give information about me that is needed. However, I understand that treatment and services cannot be conditioned upon whether I sign this agreement.

6) Signature(s) of Client or Authorized Person(s)______________________________**Date:** ___________________

7) Person Explaining Form: ____
(Name) **(Agency)** **(Phone Number)**

Place Agency Name Here:____

COORDINATION OF CARE AND SERVICES AGREEMENT

SECTION A: To be filled out by agency who originated the form.

1a) Originating Agency Representative's Name:______________________ Secure Fax:_____________ Phone:_____________

1b) Client:__ 1c) Client Date of Birth: ____

(Print Client's Full Name)

1d): Name of Organization(s) Client Being Referred to:____

____

____

SECTION B: To be filled out by medical provider or linkage personnel who will be linking the client to care after receiving referral from the original agency. Please complete Section B and fax this information back to the agency who originated this form. Please be sure to use a fax cover sheet and ensure that all fax lines are secure.

2b) Name of Person Linking Client to Care:__________________________________ 2c) Agency Name:______________________

2d)Telephone and Secure Fax Number of Person Linking Client to Care: _____________________ _______________________

Phone Secure Fax

3) Client was referred to:

Medical Provider/Agency Referred to: ____

Date of referral: ___________ Date of appointment: ___________

Confirmed attendance of appointment (Date verified): _________ Confirmation method (circle one): Phone or Fax

Other type of Service Referral: ____

Agency: _____________________ Date of referral: ___________ Date of appointment: ___________

Other type of Service Referral: ____

Agency: _____________________ Date of referral: ___________ Date of appointment: ___________

Other type of Service Referral: ____

Agency: _____________________ Date of referral: ___________ Date of appointment: ___________

Notes/Comments:

Appendix II.

SPNS Patient Navigation Client Intake Package

INSTRUCTIONS: CLIENT INTAKE FORMS

PURPOSE OF FORM: The purpose of these forms are to facilitate the client's first visit with a Patient Navigator, to establish a relationship with the client, and to assess initial barriers to linking and/or retaining in HIV medical care.

WHEN TO ADMINISTER: This form should be administered at the first intake visit or encounter with the Patient Navigator.

FORM INSTRUCTIONS:

1. **INTAKE CHECKLIST:**

   - **The *Client Intake Visit Checklist*** contains the necessary elements that should be completed at a client intake visit, also outlined in the proceeding sections. The Patient Navigator should fill this checklist out as they complete components of the intake visit. The Patient Navigator should also document the start and end time of the client intake visit. The checklist represents the core/minimum activities that should occur at a client intake visit. Additional activities or issues addressed should be documented in the notes section of this form. This document should be stored as the first page of the client intake package in the client's file.

2. **CLIENT CONTACT FORM:**

   - The ***Client Contact Information Form*** should be filled out first at the client intake visit and should be updated at each subsequent client visit. The purpose of this form is to provide the Patient Navigator with the most up-to-date client contact information including address, phone, email address and the best way to contact the client between visits. Clients should also be asked if they would like to provide contact information for up to two alternative contacts and whether these individuals can be contacted on behalf of the client. However, clients are not required to provide contact information for any alternative contacts.

   - It is important to update or confirm information on this form at **each client visit** to ensure that the Patient Navigation program has accurate information on transient clients or clients with unstable or sporadic housing situations. Furthermore, if the client wishes to provide a different address or contact method other than their permanent address, this form can be used to capture that information to maximize the Patient Navigator's ability to communicate and contact the client through their **preferred method**.

3. **CLIENT LINKAGE TO CARE PLAN:**

   - Using the ***Client Assessment Form*** (Barriers Assessment) and the ***Linkage to Care Plan (LCP)*** as resources, the Patient Navigator should use client-centered Motivational Interviewing skills to explore up to three potential barriers linking into medical care, particularly focused on attending their first or next medical appointment.

   - The Patient Navigator should record notes related to these barriers on the LCP document and explore possible solutions to overcome these barriers with the client. These plans should be documented by the Patient Navigator in the Solution/Plan to Overcome Barrier section of the form as well as client-offered target dates for resolving these barriers.

   - The Patient Navigator **should offer the client a copy of the LCP (without the client identifier on it)** at the end of the visit. If the client refuses a copy of the LCP, this should be noted on the checklist. At each subsequent visit, the Patient Navigator will conduct another barriers review and assessment using the Retention in Care Plan (RCP).

SPNS Patient Navigation <u>Intake Visit</u> Checklist

Client ID:________________________________

Date: ____________ Assigned staff member: ______________

Intake visit start:_________am/pm Intake visit end:__________am/pm

Conduct activities in the order listed on this page at the client's intake visit. Check the appropriate box, as completed:

- ☐ Date Referral Received: ____________
- ☐ Complete Client Contact Information Form
- ☐ Conduct necessary Financial Screening for Services Eligibility (Ryan White and others)
  - ☐ N/A, as financial screening completed by another provider
- ☐ Facilitate or confirm next client medical appointment. Date of appointment: _____________
- ☐ Fax back confirmed date of medical appointment to referring agency using page 2 the Coordination of Care Services Agreement form
- ☐ Conduct Initial Barriers Assessment and complete a Linkage to Care Plan (LCP) form
- ☐ Make a copy of the LCP for the client to take home that <u>does not have Client ID on it</u>
  - ☐ Client refused copy of LCP
- ☐ Offer client Partner Testing Services (if applicable)
- ☐ Provide tailored client education (list components covered):

  ____

- ☐ Complete the Client Encounter form (or ensure that all data is recorded electronically).

Please use the space below to document any notes or additional information that may not be captured above:

SPNS Patient Navigation Client Contact Information

Client ID: ______________________ Date:_____________ Staff member: _______________

Please provide your primary contact information:
Address:

____

____

Phone number:
Home:___________________
Cell: ___________________
Other: __________________

Email address: _______________________

Can you receive text messages? ________

Alternative Contact #1: Is there someone we can contact on your behalf, if we can't reach you using the information listed above?

☐ Yes ☐ No

If yes, Name and Address:

____

____

Phone number:
Home:___________________
Cell: ___________________
Other:___________________

Email address: _______________________

Can they receive text messages? ________

Alternative Contact #2: Is there a second person we can contact on your behalf, if we can't reach you using the information listed above?

☐ Yes ☐ No

If yes, Name and Address:

____

____

Phone number:
Home:___________________
Cell: ___________________
Other:___________________

Email address: _______________________

Can they receive text messages? ________

What's the best way to get in touch with you?

☐ Home phone ☐ Email

☐ Cell phone ☐ Postal Mail

☐ Other phone ☐ Alternative Contact #1

☐ Text message ☐ Alternative Contact #2

Linkage to Care Service Plan: Assessing and Addressing Barriers (PAGE 1 of 1)
(to be assessed at each client intake visit/encounter)

Client ID: ______________________ **Date: ___________________**

Client's goal:

Identify up to three barriers to achieving client's goal, and potential solutions to those barriers:

Barrier #1:

Solution/plan to overcome Barrier #1: **Target Date:**

Barrier #2:

Solution/plan to overcome Barrier #2: **Target Date:**

Barrier #3:

Solution/plan to overcome Barrier #3: **Target Date:**

Client ID:______________________________

Barriers Checklist

(Resource for Linkage to Care Plan and Retention in Care Plan)

The barriers checklist can be used to help identify most prominent barriers to linking or staying in care that the client identifies during a Patient Navigation encounter.

Check all barriers that apply:

	Transportation
	Scheduling Issues (e.g. need to work during clinic hours)
	Financial
	Child Care
	Unstable Housing
	Mental Health issues
	Substance Abuse issues
	Stigma/Social
	Issues with Current Medical Provider
	Issues with Medication Adherence/Taking HIV Medication
	Legal Issues
	Food/Nutrition Issues
	No barriers identified
	Other, please specify:___________________

SPNS Patient Navigation
Client Assessment Form

INSTRUCTIONS: PATIENT NAVIGATION CLIENT ASSESSMENT

PURPOSE OF FORM: This form should be used to assess the client's needs and barriers to linking and retaining in HIV medical care.

WHEN TO ADMINISTER THIS FORM:

- This form should be administered during **client intake** into Patient Navigation services **and**
- This form should be administered again when assessing client readiness for **transition/discharge,** which should occur **every 6 months**. For Ryan White clients, it is recommended that, if possible, this form be completed while conducting Ryan White recertification, which occurs every 6 months.

FORM INSTRUCTIONS:

1. The Patient Navigator should explain the purpose of the form to the client. The purpose of the form is to identify the client's barriers to HIV medical care at the point of Patient Navigation intake and to see if these barriers are still present upon transition/discharge from Patient Navigation services in order to assess the client's readiness to transition from services.
2. Enter the date, Client's ID, and Patient Navigator's name.
3. The form should be completed **by the client** at Patient Navigation intake and again prior to discharge from Patient Navigation services. If the client is unable to read the form, the Patient Navigator should assist the client.
4. This form should be used to help determine develop both the client's Linkage to Care Plan and to determine whether the client is ready to develop a Client Transition Plan from Patient Navigation services.
5. If the client identifies all of the same barriers prior to transition or discharge as they did at the time of intake, further assessment is needed to determine if the client is appropriate for transition and discharge from Patient Navigation services.
6. If this form is being used to assess client readiness to transition from Patient Navigation services, the Patient Navigator should fill out Section 3 with the date of the client's most recent HIV medical appointment and the date of the client's most recent HIV medication pick-up.
7. Original copies of the completed forms should be kept with the client's Patient Navigation care plan files.

The Patient Navigator should also document data from the Client Self-Assessment in the agency's electronic data system (or the state Ryan White system) along with information from the client's Linkage to Care Plan and Client Transition Plan.

PATIENT NAVIGATION CLIENT ASSSESSMENT

1. Client Information:

a. Date:	b. Client/ID:	c. Patient Navigator Name :

2. It has been OR will be difficult for me to attend my HIV medical appointments because: (Please check all boxes that apply)

	I need a more reliable source of transportation
	I am uninsured
	I have limited or no income
	I have to work during clinic hours
	Child care is not available and/or affordable for me
	My living situation is unstable
	I feel depressed, anxious or have other mental health concerns
	I am using drugs and/or alcohol
	I am confused about how to schedule appointments
	I am too busy to go to my appointments

	I feel healthy
	Going to appointments reminds me that I have HIV
	I am afraid other people will know that I have HIV
	HIV treatment won't work for me
	I want to see a different medical provider
	I forgot about my appointment
	Other:
	Other:
	Other:

Other Comments:

**3. For Patient Navigator use when used during client discharge/transition assessment:**	d. Date of Most Recent HIV Medical Visit:	e. Date of Most Recent HIV Medication Pick-Up:
**4. For Patient Navigator use when used during client discharge/transition assessment:**	f. Transition/Discharge Recommended:<br>YES NO	g. Reason:

Appendix III.

SPNS Patient Navigation
Client Visit/Encounter Package

INSTRUCTIONS: CLIENT ENCOUNTER FORMS

PURPOSE OF FORMS: The purpose of these forms is to facilitate a client encounter or visit with a Patient Navigator and to address barriers to linking and/or retaining in HIV medical care.

WHEN TO ADMINISTER: These forms should be administered at each client visit with the Patient Navigator.

FORM INSTRUCTIONS:

1. **CLIENT ENCOUNTER CHECKLIST:**

   - **The *Client Encounter Visit Checklist*** contains the necessary elements that should be completed at a client encounter. The Patient Navigator should also document the start and end time of the client intake visit. The checklist represents the core/minimum activities that should occur at each client visit. Additional activities or issues addressed should be documented in the notes section of this form. This document should be stored in the client's file.

2. **CLIENT CONTACT FORM:**

   - The ***Client Contact Information Form*** should be filled out first at the client intake visit and should be updated at each subsequent client visit. The purpose of this form is to provide the Patient Navigator with the most up-to-date client contact information including address, phone, email address and the best way to contact the client between visits. Clients should also be asked if they would like to provide contact information for up to two alternative contacts and whether these individuals can be contacted on behalf of the client.

   - It is important to update or confirm information on this form at **each client visit** to ensure that the Patient Navigation program has accurate information on transient clients or clients with unstable or sporadic housing situations. Furthermore, if the client wishes to provide a different address or contact method other than their permanent address, this form can be used to capture that information to maximize the Patient Navigator's ability to communicate and contact the client through their **preferred method**.

3. **CLIENT RETENTION IN CARE PLAN (RCP):**

   - At each client encounter or visit, the Patient Navigator will use client-centered approaches to engage the client in identifying and addressing barriers to staying in HIV care. The top portion of the Retention in Care Plan (RCP) is designed to help the client and Patient Navigator review accomplishments and barriers that have been overcome since the last encounter as well as ongoing barriers that may persist. The form is designed to facilitate a client-driven discussion identifying new barriers and ways to overcome them. The Patient Navigator should document notes on the barriers and the plan on the RCP document. Like the Linkage to Care Plan, a copy of the RCP should also be given to the client at the conclusion of the visit (without client name or ID).

4. **CLIENT ENCOUNTER FORM:**

   - After each client encounter/visit, the Patient Navigator should document relevant data from the visit in the site's electronic reporting system or in the attached Client Encounter Form or in the agency's electronic database. This form provides information on the type, method, location, and duration of services provided to the client. This de-identified data is also collected for program evaluation purposes by VDH.

SPNS Patient Navigation Encounter/Visit Checklist

Client ID:____________________ Date:_____________Assigned staff member: _______________

Intake visit start:__________am/pm Intake visit end:___________am/pm

Conduct activities in the order listed on this page at the client's intake visit. Check the appropriate box, as completed:

- ☐ Update Client Contact Information Form
- ☐ Facilitate or confirm next client medical appointment. Date of appointment: ______________
- ☐ Review client accomplishments and ongoing barriers and complete the Retention In Care Plan (RCP) form
- ☐ Make a copy of the RCP for the client to take home that does not have Client ID on it
  - ☐ Client refused copy of RCP
- ☐ Make referrals to support services to address barriers listed in RCP if applicable.
- ☐ Offer client Partner Testing Services (if applicable in encounter setting)
- ☐ Provide tailored client education (list components covered):

  ____

- ☐ Complete the Client Encounter form (or ensure data is recorded electronically).

  Please use the space below to document any notes or additional information that may not be captured above:

SPNS Patient Navigation Client Contact Information (*for update at each encounter*)

Client ID:____________ Date:_____________ Staff member: ______________

Please provide your primary contact information:

Address:

____

____

Phone number:
Home:__________________
Cell: __________________
Other:__________________

Email address: ______________________

Can you receive text messages? ________

Alternative Contact #1: Is there someone we can contact on your behalf, if we can't reach you using the information listed above?

☐ Yes ☐ No

If yes, Name and Address:

____

____

Phone number:
Home:__________________
Cell: __________________
Other:__________________

Email address: ______________________

Can they receive text messages? ________

Alternative Contact #2: Is there a second person we can contact on your behalf, if we can't reach you using the information listed above?

☐ Yes ☐ No

If yes, Name and Address:

____

____

Phone number:
Home:__________________
Cell: __________________
Other:__________________

Email address: ______________________

Can they receive text messages? ________

What's the best way to get in touch with you?

☐ Home phone ☐ Email

☐ Cell phone ☐ Postal Mail

☐ Other phone ☐ Alternative Contact #1

☐ Text message ☐ Alternative Contact #2

Retention in Care Service Plan: Overcoming Barriers (PAGE 1 of 2)
(to be assessed at each subsequent client visit/encounter)

Client ID: ______________________ **Date:** ___________________

Accomplishments and Resolutions:
Use the space below to document resolution of barriers from previous appointment and client accomplishments.

Ongoing Barriers:
Use the space below to document any ongoing or persistent barriers that may not have been resolved since the previous appointment.

Retention in Care Service Plan: Overcoming Barriers (PAGE 2 of 2)
(to be assessed at each subsequent client visit/encounter)

Client ID: ________________________ **Date: _____________________**

Client's goal:

Identify up to three barriers to achieving client's goal, and potential solutions to those barriers:

Barrier #1:

Solution/plan to overcome Barrier #1: **Target Date:**

Barrier #2:

Solution/plan to overcome Barrier #2: **Target Date:**

Barrier #3:

Solution/plan to overcome Barrier #3: **Target Date:**

Appendix IV.

SPNS Patient Navigation
Client Transition Package

SPNS Patient Navigation
Client Assessment Form

INSTRUCTIONS: PATIENT NAVIGATION CLIENT ASSESSMENT

PURPOSE OF FORM: This form should be used to assess the client's needs and barriers to linking and retaining in HIV medical care.

WHEN TO ADMINISTER THIS FORM:

- This form should be administered during **client intake** into Patient Navigation services
  and
- This form should be administered again when assessing client readiness for **transition/discharge,** which should occur **every 6 months**. For Ryan White clients, it is recommended that, if possible, this form be completed while conducting Ryan White recertification, which occurs every 6 months.

FORM INSTRUCTIONS:

1. The Patient Navigator should explain the purpose of the form to the client. The purpose of the form is to identify the client's barriers to HIV medical care at the point of Patient Navigation intake and to see if these barriers are still present upon transition/discharge from Patient Navigation services in order to assess the client's readiness to transition from services.

2. Enter the date, Client's ID, and Patient Navigator's name.

3. The form should be completed **by the client** at Patient Navigation intake and again prior to discharge from Patient Navigation services. If the client is unable to read the form, the Patient Navigator should assist the client.

4. This form should be used to help develop both the client's Linkage to Care Plan and to determine whether the client is ready to develop a Client Transition Plan from Patient Navigation services.

5. If the client identifies all of the same barriers prior to transition or discharge as they did at the time of intake, further assessment is needed to determine if the client is appropriate for transition and discharge from Patient Navigation services.

6. If this form is being used to assess client readiness to transition from Patient Navigation services, the Patient Navigator should fill out Section 3 with the date of the client's most recent HIV medical appointment and the date of the client's most recent HIV medication pick-up.

7. Original copies of the completed forms should be kept with the client's Patient Navigation care plan files.

The Patient Navigator should also document data from the Client Self-Assessment in the agency's electronic data system (or the state Ryan White system) along with information from the client's Linkage to Care Plan and Client Transition Plan.

PATIENT NAVIGATION CLIENT ASSSESSMENT

1. Client Information:

a. Date:	b. Client:	c. Patient Navigator Name :

2. It has been OR will be difficult for me to attend my HIV medical appointments because: (Please check all boxes that apply)

	I need a more reliable source of transportation
	I am uninsured
	I have limited or no income
	I have to work during clinic hours
	Child care is not available and/or affordable for me
	My living situation is unstable
	I feel depressed, anxious or have other mental health concerns
	I am using drugs and/or alcohol
	I am confused about how to schedule appointments
	I am too busy to go to my appointments

	I feel healthy
	Going to appointments reminds me that I have HIV
	I am afraid other people will know that I have HIV
	HIV treatment won't work for me
	I want to see a different medical provider
	I forgot about my appointment
	Other:
	Other:
	Other:

**Other Comments:**

**3. For Patient Navigator use when used during client discharge/transition assessment:**	d. Date of Most Recent HIV Medical Visit:	e. Date of Most Recent HIV Medication Pick-Up:
**4. For Patient Navigator use when used during client discharge/transition assessment:**	f. Transition/Discharge Recommended:<br>YES NO	g. Reason:

INSTRUCTIONS: CLIENT TRANSITION PLAN

PURPOSE OF FORM: The purpose of this form is to develop an HIV care transition plan with clients transitioning from Patient Navigation services to case management, self-management, or other services.

WHEN TO ADMINISTER THIS FORM: This form should be administered when the client has been determined eligible for transition or discharge from Patient Navigation services. Clients should be assessed for transition/discharge from Patient Navigation services at least **every six months using the Client Assessment form (attached)**. The following should be considered during the transition assessment process in consultation between the Patient Navigator and client:

- Attendance at HIV medical care appointments (2 or more medical appointments within 6 months)
- Verification of medication pick-up
- An assessment of barriers and needs documented on the **Client Assessment form** (attached) and Client Linkage and Retention in Care Plans

FORM INSTRUCTIONS:

1. **Enter client information:**
   a. Enter Transition Plan date (today's date).
   b. Enter Client ID (agency-specific).
   c. Enter Patient Navigator name.
   d. Enter date of client's most recent HIV medical appointment.
   e. Enter date of client's most recent HIV medication pick-up.

2. **Transition Service Information:**
   Indicate the services that the client will transition to after discharge from Patient Navigation services. List the name of the agency and provider (if applicable). More than one box may be checked if client will be working with both a medical and non-medical case manager after discharge.

3. **Summary of Resolved and Unresolved Needs/Barriers**:
   a. For each "Indicated Need/Barrier", summarize the issues that have been resolved during the client's enrollment in Patient Navigation services in the "Resolved" column.
   b. For each "Indicated Need/Barrier", summarize the issues that will still require action after the client's discharge from Patient Navigation in the "Unresolved" column.
   c. Enter the individual responsible for taking action on any unresolved issues in the "Responsibility" column.

4. **Transition Plan Agreement:**
   The client, Patient Navigator, and Patient Navigator supervisor should sign to indicate approval of Transition Plan prior to discharge from Patient Navigation services.

   If client will be transitioned to case management or other services, the case manager or other transition personnel should review and sign this plan with the client and Patient Navigator present. If it is not possible to have the case manager or transition personnel present, the Patient Navigator should fax this form to the case manager or transition personnel for review. The case manager or transition personnel should sign the form and fax it back to the Patient Navigator.

CLIENT TRANSITION PLAN

1. Client Information:

a. Date:	b. Client/ID:	c. Patient Navigator (PN) Name :

d. Date of Most Recent Client HIV Medical Visit:	e. Date of Most Recent HIV Medication Pick-Up:

2. Transition Service Information. Client will transition to the following services:

☐ a. Medical Case Management Agency Name & Contact: :____

☐ b. Non-Medical Case Management Agency Name & Contact: :____

☐ c. Other Agency Name & Contact: :____

☐ d. Self-Management

3. Summary of Resolved and Unresolved Needs/Barriers:

Need/ Barrier	a. Resolved	b. Unresolved	c. Plan / Responsibility
Clinical/ Medical			
Medication Adherence			
Mental Health/ Substance Abuse			
Transportation or Logistics			

Need / Barrier	a. RESOLVED	b. UNRESOLVED	c. Plan / Responsibility
Housing			
Employment/ Financial			
Cultural/ Linguistic			
HIV Education/ Prevention			
Insurance/ Benefits			
Other			

4. Transition of Care Plan Agreement: Please sign and date. I have read, understand, and agree with the above transition plan.

*Client or guardian:____ Date:___/ _/_____

*Patient Navigator:____ Date:___/ _/_____

*Patient Navigator Supervisor:______________________________________ Date:___/ _/_____

If client will be transitioned to case management or other services, the case manager or other transition personnel should review this plan **with** the client and PN and sign below. If this is not possible, the PN should fax a copy of this form to the transition personnel or case manager for review. The case manager or transition personnel should sign this form and fax it back to the PN.

Case Manager/Other Transition Personnel:__________________________ Date:___/ _/_____

*If more space is needed to document barriers, needs, or issues related to transition, please use this additional page.

Need/ Barrier	a. RESOLVED	b. UNRESOLVED	c. Plan / Responsibility

Appendix V.

SPNS Patient Navigation Client Discharge Form

PATIENT NAVIGATION DISCHARGE FORM

CLIENT ID:

PATIENT NAVIGATION DISCHARGE INFORMATION

a. Patient Navigator Name:	b. Discharge Date:

c. Discharge Reason:

☐ Service Completion ☐ Client request ☐ Deceased
☐ Unable to contact ☐ Transferring to another Patient Navigator ☐ Incarcerated
☐ Patient Navigator request ☐ Moved out of service area ☐ Other (specify):________

d. Discharge Service Level (select all that apply):

☐ Self-Management ☐ Other Services
☐ Non-Medical Case Management ☐ Medical Case Management
☐Other Patient Navigator (for transfers only)

e. Name of Agency Providing Case Management or Other Services(if applicable):

CLIENT INFORMATION

f. Date of Most Recent Medical Visit	g. Date of Most Recent HIV Medication Pick-Up

h. Current Housing Status:

☐ Stable/Permanent ☐ Unstable ☐ Temporary ☐Unknown

i. Current Insurance Status (select all that apply):

☐ Private ☐ Medicare ☐ Medicaid ☐ No insurance ☐ Unknown ☐ Other (specify):

CLIENT DISCHARGE APPROVAL

Patient Navigator:____ Date:___/___/_____

Patient Navigator Supervisor:_____________________________________ Date:___/___/_____

Appendix VI.

Division of Disease Prevention Guidelines for Providing In-Home OraQuick® Tests

Appendix VI.

DDP GUIDELINES FOR PROVIDING IN-HOME ORAQUICK® TESTS

The Virginia Department of Health (VDH) prefers that people receive HIV testing in a setting that can provide appropriate pre and post-test counseling, answer questions, and link the patient to care and support services as needed. There are times, however, when individuals are unable or unwilling to present for testing. The in-home test provides an additional option for ensuring that individuals are tested and learn their HIV status. The in-home tests are only to be used for individuals who are 17 and older.

As appropriate, counselors should first encourage people to conduct an HIV test on-site. Individuals who are offered in-home tests should be high-risk individuals who are unable or unwilling to get tested through an established medical, community or outreach location. In-home tests may also be provided to individuals who identify others in their social networks who are at risk for HIV, but unwilling to test on-site or have challenges related to transportation/distance from a test site.

Ideally, test reporting information shall be requested for each in-home test. Each testing site needs to report the number of tests distributed and, if possible, obtain basic demographic information (race, gender, age, risk if known) and test results. This collection may occur in a number of ways (online, by phone, in person). See the attachment for a template data collection form that may be individualized by agency.

Testing sites need to ensure that in-home test kit recipients have contact information for counseling and follow up testing. While each kit already contains a national hotline number set up by OraSure Technologies, Inc, participating agencies must also include additional referral information, such as the VDH Hotline, the agency contact number, and instructions for next steps based on the test result.

Sites should seek a commitment from people to actually use the test, whether or not they report back to the agency, as the tests are expensive. Clients who receive test kits for distribution should be advised that the test kits may not be sold and cannot be returned to drug stores for cash as the boxes are not coded for individual sale. Agencies should also set an initial limit on the number of test kits that individuals may receive.

Clients should be counseled that, if they plan to use the test to verify the status of sex partners prior to engaging in sex, they need to be aware that:

- the test may not pick up infection that occurred within the last three months;
- they should not use a negative test result as an indicator that it is safe to engage in unprotected sex; and
- they need to consider the implications of a sex partner learning their HIV status in the client's home or location where they are engaging in sex (e.g., reaction to a test result, need for additional assistance, etc).

Innovative approaches, such as using Skype or i-Chat to provide pre and post counseling, should be discussed and approved by the local or state health department or HIV certified testing program. Innovative approaches, such as using Skype or i-Chat to provide pre and post counseling, should be discussed and approved by the DDP contract monitor in advance. Testing sites interested in providing an incentive for the in-home test should contact their contract monitor for guidance. A brief survey will be distributed by DDP to contractors and clients to assess this new testing.

Appendix VII.

Service Categories for Patient Navigation Data Collection

Appendix VII.
VIRGINIA DEPARTMENT OF HEALTH (VDH)
SERVICE CATEGORIES FOR PATIENT NAVIGATION DATA COLLECTION

VDH provided specific service categories for Patient Navigation data collection. VDH also designed and made available an electronic reporting system to two of the three SPNS demonstration sites. The third site utilized their existing Ryan White CAREWare electronic reporting system. Regardless of the electronic system used, the collection of the following data was required:

Coordination of Care and Services Agreement Form Data:

- Date of Signed CCSA form
- Medical/Agency Referred To
- Date of Referral to medical agency
- Date of Medical Appointment
- Date confirmed attendance at medical appointment
- Confirmation Method (phone, fax, etc)

Intake and Encounter Data: Enter data for ALL PN encounters with or on behalf of client.

- Who referred the client to the PN (intake only)
- Date Client Referred to PN (intake only)
- Date PN first contacted client (intake only)
- Type of Contact: With Client or On Behalf of Client
- Method of Contact:
  - Face to face
  - Phone
  - Text message
  - Email
  - Social Network Site
  - In a Group
  - Postal Mail
  - Administrative, Paperwork, Research
  - Other
- Client Encounter Duration in minutes
- Where was PN service provided
  - Clinic
  - CBO
  - Home
  - Other
- PN Services provided/or referred to Client:
  - Medical
  - Financial
  - Housing
  - Substance Abuse Treatment
  - Mental Health Treatment
  - Insurance/Benefits
  - Initial Screening with navigator

    - Case management
    - Attended appointment
    - Emotional Support
    - Other

Discharge Data:

- Date of client discharged from PN services
- Discharge Reason
    - Service Completion
    - Client Request
    - Client Deceased
    - Unable to Contact
    - PN Transfer
    - Client Incarcerated
    - Patient Navigator Request
    - Moved out of Area
    - Other
- Discharge Service Level
    - Self Management
    - Medical Case Management
    - Non-Medical Case Management
    - Other Patient Navigator
    - Unknown/Undetermined
    - Other Services
    - Not Applicable
- Current Housing Status at Discharge
    - Stable
    - Unstable
    - Temporary
    - Unknown
- Current Insurance Status at Discharge
    - Private
    - Medicare
    - Medicaid
    - Unknown
    - Other

Appendix VIII.

Sample Patient Navigator Job Description

Appendix VIII.

SAMPLE JOB DESCRIPTION

Position Purpose: Facilitate linkage and retention in HIV medical care for HIV- individuals

Objectives of Position:

To support client linkage and retention in HIV care for a period of 3-12 months by:

I. Facilitating entry into medical care for newly-diagnosed HIV-positive individuals, individuals who have been lost to HIV medical care, individuals "at risk" of dropping out of medical care, and individuals who have never entered the care system.

II. Providing client-centered counseling to assess and address client barriers to linking and retaining in HIV medical care.

III. Facilitating client linkage to medical care and needed support services and provide necessary follow up to support client retention in care.

IV. Providing client-centered education related to HIV disease, medical care, medications, and risk-reduction.

V. Using Motivational Interviewing techniques to engage and empower client toward self-management.

VI. Transitioning clients to community services (such as case management) or self-management of their HIV medical care.

VII. Building relationships and communicating with providers and community agencies to accelerate and enhance client linkage and retention in care.

VIII. Facilitating with the early identification of individuals with HIV/AIDS by providing referrals to HIV testing for contacts and partners of clients and the distribution of in-home HIV test kits in circumstances where site referrals are not feasible.

Minimum Qualifications:

- Familiar with client-centered approaches to service delivery
- Strong skills in networking with HIV services providers, the community and high-risk populations
- Strong interviewing and oral communication skills
- Ability to provide culturally competent and sensitive health and risk reduction educational messages
- Ability to work in team setting
- Strong time management skills
- Knowledge of community resources and the ability to develop new linkages in the community
- Familiarity of health care delivery/services in particular service area

Preferred Qualifications:

- Bachelors Degree in health related field **<u>or</u>** a minimum of three years experience working within a health care delivery system, substance abuse, mental health, with incarcerated individuals or in HIV service provision

Appendix IX.

Patient Navigator Training

Appendix IX.

PATIENT NAVIGATOR TRAINING

The success of a Patient Navigation intervention relies heavily on the abilities of Patient Navigators including their capacity to build working relationships, solve various problems creatively and effectively, direct clients to resources, and to manage information. Navigators should also possess compassion, intelligence, great communication skills, cultural sensitivity and ingenuity. Appropriate and effective initial and ongoing training is critical to the success of the Patient Navigation intervention.

As part of Virginia's SPNS Systems Linkages Initiative, the Virginia Department of Health contracted with a Pennsylvania/Mid Atlantic AIDS Education and Training Center performance site at the Inova Juniper Program in Springfield, Virginia to provide Patient Navigation training. The Institute for Drug and Alcohol Studies (IDAS) at the Virginia Commonwealth University in Richmond, Virginia was contracted to provide Motivational Interviewing and Fidelity Monitoring activities for Patient Navigator.

Patient Navigators were expected to attend and participate in monthly web meetings, quarterly in-person trainings, and technical assistance sessions on various topics selected by the training contractor in coordination with the Virginia Department of Health (VDH) and IDAS. The topics were designed to meet Core Competencies while also taking into consideration input from Patient Navigators and their supervisors. Some training components were offered in web-based format and others were required to be conducted in-person. Below is an outline of the Core Competencies and accompanying Training Requirements for the SPNS Patient Navigation Training Plan. Each Patient Navigator was expected to complete the following trainings (participating in an ongoing basis). Patient Navigation sites were expected to keep documentation of trainings completed or in-progress in their monthly reports to VDH and in their program files.

Core Competencies and Expected Skills

**Core Competency**	**Expected Skills:**
The Role and Practices of the Patient Navigator	• Discuss the definition of a Patient Navigator<br>• Define the role of a Patient Navigator, understand the population served, and communicate the relationship with the rest of the healthcare system<br>• Identify appropriate written and web-based resources for clients regarding all aspects of HIV<br>• Interact with other Patient Navigators in the program to establish rapport and referral relationships

Core Competency	Expected Skills:
HIV Facts: HIV Disease Basics: Medical, life cycle, treatment and adherence	• Understand the spectrum of HIV disease<br>• Discuss HIV antibody testing options including legal implications, process of testing, and meaning of results<br>• Describe partner counseling and referral services in the Commonwealth of Virginia<br>• Describe effective techniques for providing HIV risk assessment and reduction<br>• Discuss implications of HIV infection as experienced by an HIV-infected person
Introduction of Critical Concepts: Motivational Interviewing, Client Provider Relationships, Exploring Barriers to Care	• Understand motivational interviewing and ways to apply these technique to linking clients to care<br>• Practice using open-ended questions, affirmations, reflections, and summaries when working with a client<br>• Describe the four processes of motivational interviewing (Engaging, Focusing, Evoking, Planning)<br>• Recognize client barriers in negotiating healthcare systems and strategies to assist them
Field Safety Overview	• Understand field safety guidelines and protocol.<br>• Describe professional etiquette on home and field visits<br>• Plan safe home visits and strategic exit plans<br>• Awareness of importance of informing colleagues of visit and the distance/time it takes to travel.
Home Testing Kits Protocol, Guidelines, and Guidance: The Basic lab results, rapid methodologies, procedures for referring, protocol follow-up	• Describe the guidelines and guidance<br>• Understand how to read kit results<br>• Explain the protocol for distribution of the home kits

Core Competency	Expected Skills:
Motivational Interviewing (MI) Intensive	• Practice, apply, and integrate through role-play and small group exercise:<br>• Open-ended Questions<br>• Affirmations<br>• Reflections<br>• Summaries<br>• 4 processes Engaging, Focusing, Evoking, Planning<br>• Explore the importance of using motivational interviewing skills to help clients to explore and resolve ambivalence<br>• Understand the expectations of Fidelity Monitoring (taping)
HIV Prevention Counseling: The Fundamentals and Home Testing Hands-On	• Identify strategies to support clients in making behavior changes that will reduce the risk of acquiring or transmitting HIV<br>• Demonstrate through practice sessions the six steps of prevention counseling<br>• Support the client in making a decision about testing and preparing for the result<br>• Assist the client who tests to begin to integrate the result emotionally, behaviorally, and socially<br>• Demonstrate and instruct appropriate usage of the home kit
Cultural Competency Overview	• Recognize the importance of health literacy to the client experience<br>• Understand client attitudes towards health care and their provider based on cultural background<br>• Recognizing system-level strategies and tools to close the disparity gap in health care
Linkage to Care and Active Referral	• Understand the meaning and significance of “linkage to care”<br>• Demonstrate the utility of motivational interviewing to facilitate linkage<br>• Define the active referral process and how it supports linking the client to care

Core Competency	Expected Skills:
Disclosure & Stigma	• Recognize and evaluate the effect of stigma on the decisions of a client<br>• Assist the client to identify issues related to stigma in their own life<br>• Support a client in making decisions regarding disclosure<br>• Utilize motivational interview techniques to help client explore and role play disclosure scenarios
Patient Navigation: Client Perspectives	• Understand views and concerns of clients regarding what is effective in helping link to care, adhere to medication, etc<br>• Explore and develop alternative methods for engaging the client
Self-Care and Managing Stress Related to the Patient Navigator Role	• Identify Stress Management techniques<br>• Understand personal stressors and cues<br>• Develop a plan to manage one's own stress by using the discussed techniques
Sexually Transmitted Infections (STIs)	• Understand the different types of STIs<br>• Define the signs and symptoms of STIs<br>• Identify STI treatment strategies
Recognizing Mental Health, Substance Abuse, Psychosocial Issues	• Identify mental health and substance abuse issues that may face the client<br>• Describe effective techniques to address these issues<br>• Explore resources and referral sites available in the community to assist clients

Core Competency	Expected Skills:
Dealing with Difficult Clients	• Identify behaviors that make a client challenging and strategies to address these issues<br>• Develop an understanding that behaviors of clients are usually not about the Patient Navigators (or that “you are not the target”)<br>• Identify one’s reactions and methods to control them in dealing with a difficult client
Additional follow-up trainings based on the topics presented and the needs and input of the group	Web meeting/In-Person
Collaborations with Other Organizations	Through the training process, collaborations with other organizations are likely to develop. These relationships may lead to co-facilitating additional trainings and developing new ideas for new training topics.<br><br>If there are Patient Navigators at these organizations, it is valuable to consider periodic, day-long, in-person training facilitated by both or all organizations involved. These events allow Patient Navigators working under different programs to share experiences, develop skills, and learn about resources.

Core Competency Trainings, Methods and Timeframes

Core Competency/Training Requirement:	Training Method:	Training Timeframe:
The Role and Practices of the SPNS Patient Navigator	In-Person, One Day	Within first 1 month of hire or program start
HIV Facts: HIV Disease Basics: Medical, life cycle, treatment and adherence	In-Person, One Day (with a recorded option available if needed)	Within first 1 month of hire or program start
Introduction of Critical Concepts: Motivational Interviewing, Facilitating the Client Provider Relationships, Exploring Barriers to Care	In-Person, One Day	Within first 1 month of hire or program start
Field Safety Overview	Web meeting	Within first 3 month of hire or program start

Core Competency/Training Requirement:	Training Method:	Training Timeframe:
Home Testing Kits Protocol/Guidelines and Guidance: The Basic lab results, rapid methodologies, procedures for referring, protocol follow-up	Web meeting	Within first 6 months of hire or program start
Motivational Interviewing (MI) Intensive	In Person, Two Day	Within first 6 months of hire or program start
HIV Prevention Counseling: The Fundamentals and Home Testing Hands-On	In-Person	Within first 6 months of hire or program start
Cultural Competency Overview	Web meeting	Within first 6 months of hire or program start
Linkage to Care and Active Referrals	Web meeting	Within first 6 months of hire or program start
Disclosure & Stigma	Web meeting	Within first 6 months of hire or program start
Patient Navigation: Client Perspectives	Web meeting	Within first year of hire or program start
Self-Care and Managing Stress Related to the Patient Navigator Role	Web meeting/In-Person (depending on the needs of the Patient Navigators)	Within first year of hire or program start
Sexually Transmitted Infections (STIs)	Web meeting	Within first year of hire or program start
Recognizing Mental Health, Substance Abuse, Psychosocial Issues	Web meeting	Within first year of hire or program start
Dealing with Difficult Clients	Web meeting	Within first year of hire or program start
Additional follow-up trainings based on the topics presented and the needs and input of the group	Web meeting/In-Person	

Motivational Interviewing & Fidelity Monitoring

An essential component of the Patient Navigator training was Motivational Interviewing. Motivational Interviewing is a collaborative conversation to strengthen a person's own motivation for and commitment to change. The Virginia Department of Health worked with the Virginia Commonwealth University's Institute for Drug and Alcohol Studies to train, monitor, and evaluate Patient Navigator's use of Motivational Interviewing skills to support client linkage and retention in HIV care. Patient Navigators audiotape client sessions, which are then reviewed by evaluation

staff using the Motivational Interviewing Treatment Integrity Code. This coding system guides individual and group feedback to PNs. The initial focus is on the establishment of MI skills with shifts towards protocol adherence and skill maintenance. Patient Navigators have found it challenging to utilize Motivational Interviewing techniques when addressing client issues that require immediate attention. Patient Navigators that have historically used different models of care have found it difficult to transition into the Motivational Interviewing style of interaction. Ongoing training and support are integral to the continued success of the Motivational Interviewing – based model.

Appendix X.

Motivational Interviewing Summary

Appendix X.

MOTIVATIONAL INTERVIEWING (MI) SUMMARY

Background:
Motivational Interviewing was initially developed by William Miller and Stephen Rollnick[1] for working with alcohol abusers. Over the past 20 years, wide application in a variety of behavioral domains and patient populations occurred. Currently, MI is used in a variety of settings (e.g., dietary and medication compliance, smoking cessation) and a range of patient conditions (e.g., hypertension, asthma, insulin dependent diabetes, eating disorders). There are over 150 empirically based clinical studies in support of MI.

Motivational Interviewing is a collaborative, client-centered, directive method for enhancing intrinsic motivation to change by exploring and resolving ambivalence. The goal is to *create and amplify discrepancy* between present behavior and broader goals.

MI is based on three key elements: collaboration between the therapist and the client; evoking the client's ideas about change; and emphasizing the autonomy of the client.

• Collaboration: Collaboration is a partnership between the therapist and the client. Collaboration builds rapport and facilitates trust in the helping relationship. Although they may see things differently, the therapeutic process is focused on mutual understanding, not the therapist being right.

• Evocation: This MI approach draws out the individual's own thoughts and ideas. The overall belief is that lasting change is more likely to occur when the client discovers their own reasons and determination to change. The therapist's job is to elicit the person's own motivations and skills for change, not to tell them what to do or why they should do it.

• Autonomy: Motivational Interviewing recognizes that the true power for change rests within the client. Ultimately, it is up to the individual to follow through with making changes happen.

There are four distinct principles that guide the practice of MI.

• Express Empathy: This approach provides the basis for clients to be heard and understood, and in turn, clients are more likely to honestly share their experiences in depth. The process of expressing empathy relies on the client's experiencing the counselor as able to see the world as they (the client) sees it.

• Support Self-Efficacy: A client's belief that change is possible (self-efficacy) is needed to instill hope about making those difficult changes. In Motivational Interviewing, counselors support self-efficacy by focusing on previous successes and highlighting skills and strengths that the client already has.

• Roll with Resistance: PNs avoid eliciting resistance by not confronting the client and when resistance occurs, they work to de-escalate and avoid a negative interaction, instead "rolling with it".

• Develop Discrepancy: When clients recognize that their current behaviors place them in conflict with their values or interfere with accomplishment of self-identified goals, they are more likely to experience increased motivation to make important life changes.

The basic approaches used in MI are Open Ended Questions, Affirmations, Reflections, and Summaries.

• Open-ended questions are those that are not easily answered with a "yes/no" or short answer. Open-ended questions invite elaboration and thinking more deeply about an issue.

• Affirmations are statements that recognize client strengths. They assist in building rapport and in helping the client see themselves in a different, more positive light.

• Reflections help the client feel that the PN understands the issues from their perspective. Reflections are also integral in guiding the client toward change. There are several levels of reflection ranging from simple to more complex.

• Summaries are a special type of reflection where the therapist recaps what has occurred in all or part of a counseling session(s). Summaries communicate interest, understanding and call attention to important elements of the discussion.

Major focuses of MI:

- Client engagement
- Exploring ambivalence
- Promoting empowerment and self-efficacy
- Collaboration between client and provider

Training:

SPNS PNs participated in the following basic training in MI:

- Online Training Modules
- Full day of in-person MI training
- 2 day experiential training with all PNs with assessment of basic skill mastery

----

Sources:

Center for Substance Abuse Treatment (1999). Enhancing Motivation for Change in Substance Abuse Treatment. Treatment Improvement Protocol (TIP) 35. Rockville, MD: Substance Abuse and Mental Health Services Administration, Center for Substance Abuse Treatment.

Miller, W.R. & Rollnick, S. (2009). Ten things that Motivational Interviewing is not. Behavioural and Cognitive Psychotherapy, 37, 129F140.

Miller, W. R., & Rollnick, S. (2002). Motivational Interviewing: Preparing people for change. Guilford press.

Appendix XI.

Process Map for Making Active Referrals to Patient Navigation Programs

Appendix XI. Active Referrals to HIV Care Process Map

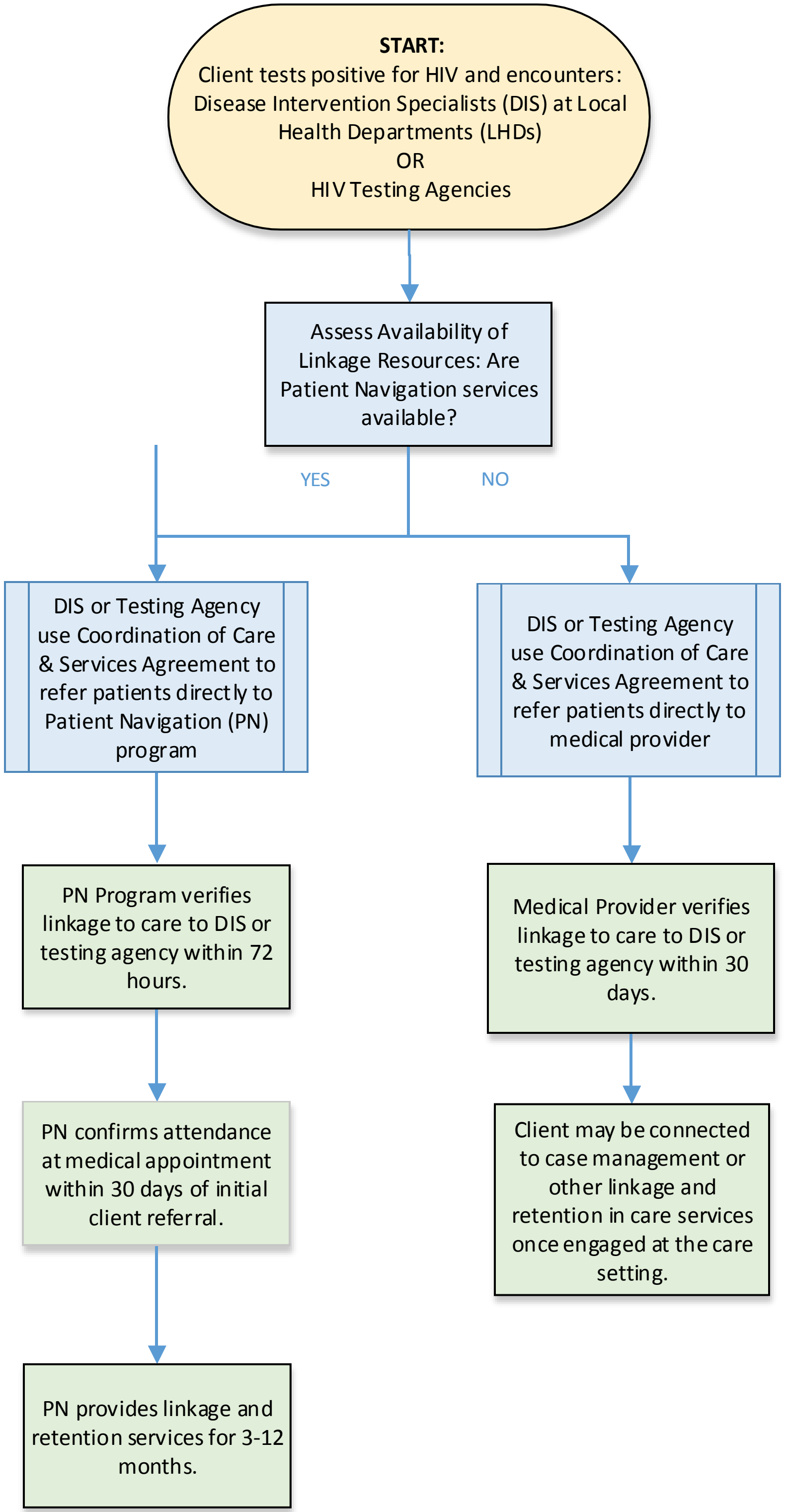

Appendix XII.

Process Map for Patient Navigation Intervention

Appendix XII. Patient Navigation Intervention Process Map

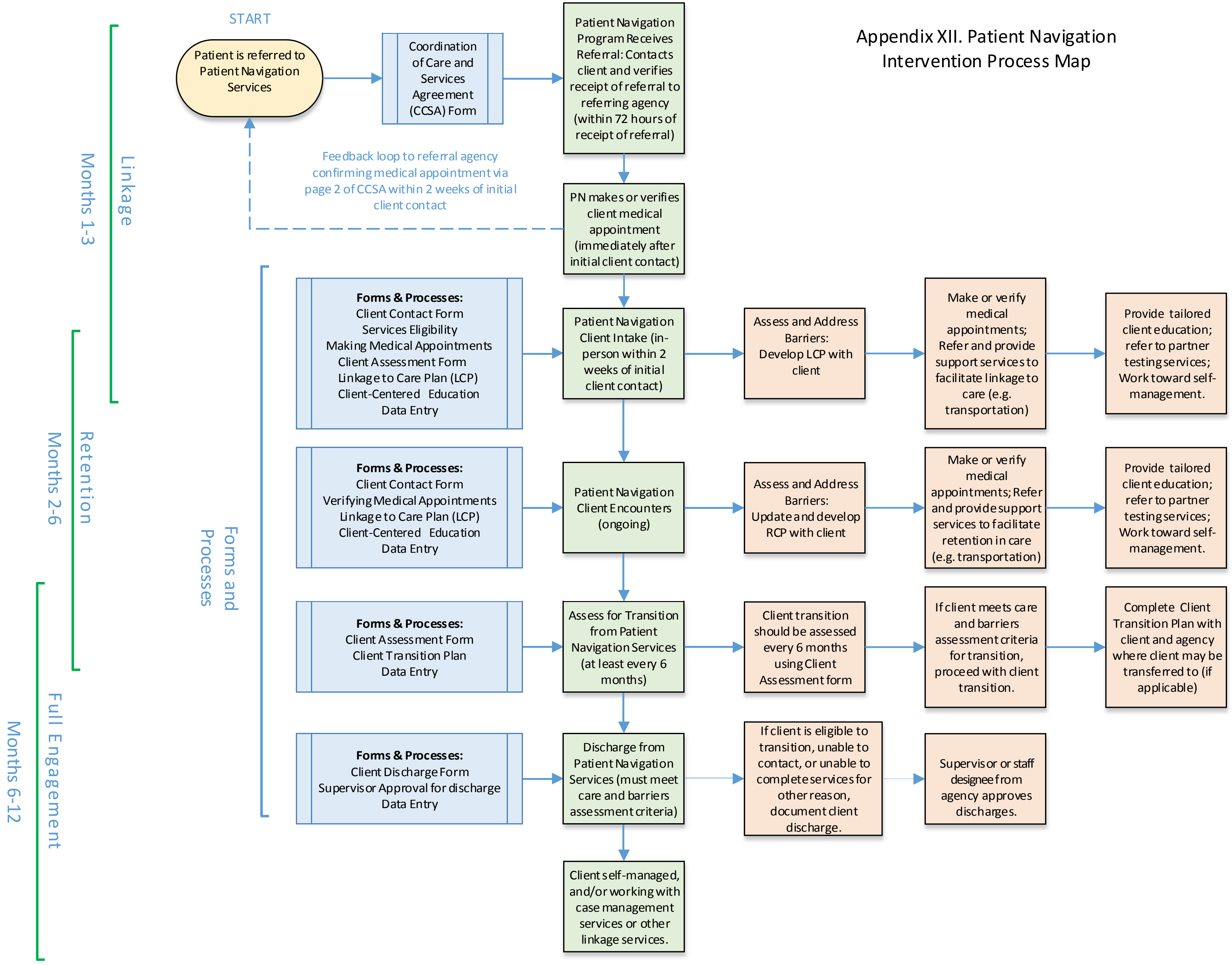

Supplement: S2 Text — (PDF) [file pmed.1003418.s005.pdf]
